# Mutational variant allele frequency profile as a biomarker of response to immune checkpoint blockade in non-small cell lung Cancer

**DOI:** 10.1186/s12967-024-05400-7

**Published:** 2024-06-18

**Authors:** Ruyun Gao, Ning Lou, Lin Li, Tongji Xie, Puyuan Xing, Le Tang, Jiarui Yao, Xiaohong Han, Yuankai Shi

**Affiliations:** 1 Department of Medical Oncology, National Cancer Center/National Clinical Research Center for Cancer/Cancer Hospital, Beijing Key Laboratory of Clinical Study On Anticancer Molecular Targeted Drugs, Chinese Academy of Medical Sciences & Peking Union Medical College, No. 17 Panjiayuan Nanli, Chaoyang District, Beijing, 100021 China; 2 Department of Clinical Laboratory, National Cancer Center/National Clinical Research Center for Cancer/Cancer Hospital, Beijing Key Laboratory of Clinical Study On Anticancer Molecular Targeted Drugs, Chinese Academy of Medical Sciences & Peking Union Medical College, No. 17 Panjiayuan Nanli, Chaoyang District, Beijing, 100021 China; 3 Department of Pathology, National Cancer Center/National Clinical Research Center for Cancer/Cancer Hospital, Beijing Key Laboratory of Clinical Study On Anticancer Molecular Targeted Drugs, Chinese Academy of Medical Sciences & Peking Union Medical College, No. 17 Panjiayuan Nanli, Chaoyang District, Beijing, 100021 China; 4 Clinical Pharmacology Research Center, Peking Union Medical College Hospital, State Key Laboratory of Complex Severe and Rare Diseases, NMPA Key Laboratory for Clinical Research and Evaluation of Drug, Beijing Key Laboratory of Clinical PK & PD Investigation for Innovative Drugs, Chinese Academy of Medical Sciences & Peking Union Medical College, Beijing, 100730 China

**Keywords:** Immunotherapy, Immune checkpoint inhibitor, Biomarker, Non-small cell lung cancer, Mutation, Variant allele frequency

## Abstract

**Introduction:**

Identifying new biomarkers for predicting immune checkpoint inhibitors (ICIs) response in non-small cell lung cancer (NSCLC) is crucial. We aimed to assess the variant allele frequency (VAF)-related profile as a novel biomarker for NSCLC personalized therapy.

**Methods:**

We utilized genomic data of 915 NSCLC patients via cBioPortal and a local cohort of 23 patients for model construction and mutational analysis. Genomic, transcriptomic data from 952 TCGA NSCLC patients, and immunofluorescence (IF) assessment with the local cohort supported mechanism analysis.

**Results:**

Utilizing the random forest algorithm, a 15-gene VAF-related model was established, differentiating patients with durable clinical benefit (DCB) from no durable benefit (NDB). The model demonstrated robust performance, with ROC-AUC values of 0.905, 0.737, and 0.711 across training (n = 313), internal validation (n = 133), and external validation (n = 157) cohorts. Stratification by the model into high- and low-score groups correlated significantly with both progression-free survival (PFS) (training: ***P*** < 0.0001, internal validation: ***P*** < 0.0001, external validation: ***P*** = 0.0066) and overall survival (OS) (n = 341) (***P*** < 0.0001). Notably, the stratification system was independent of PD-L1 (***P*** < 0.0001) and TMB (***P*** < 0.0001). High-score patients exhibited an increased DCB ratio and longer PFS across both PD-L1 and TMB subgroups. Additionally, the high-score group appeared influenced by tobacco exposure, with activated DNA damage response pathways. Whereas, immune/inflammation-related pathways were enriched in the low-score group. Tumor immune microenvironment analyses revealed higher proportions of exhausted/effector memory CD8 + T cells in the high-score group.

**Conclusions:**

The mutational VAF profile is a promising biomarker for ICI therapy in NSCLC, with enhanced therapeutic stratification and management as a supplement to PD-L1 or TMB.

**Supplementary Information:**

The online version contains supplementary material available at 10.1186/s12967-024-05400-7.

## Introduction

Immune checkpoint inhibitors (ICIs) have demonstrated unprecedented clinical success in treating non-small cell lung cancer (NSCLC), particularly in patients with advanced disease [1, 2]. The therapeutic landscape of NSCLC has been transformed by ICIs targeting the programmed death 1 (PD-1)/programmed death-ligand 1 (PD-L1) or cytotoxic T-lymphocyte antigen 4 (CTLA-4) [3]. Despite these advances, some patients who initially respond to ICIs do not maintain long-lasting benefits [4, 5]. Consequently, identifying predictive biomarkers for durable responses to ICIs is a critical challenge in contemporary clinical practice.

Although several effective biomarkers, such as PD-L1 expression [1, 2, 6], microsatellite instability (MSI)/mismatch-repair deficiency (dMMR) [7], and tumor mutation burden (TMB) [8], have been proposed, these markers have deficiencies. NSCLC exhibits a low prevalence of MSI and dMMR (< 5%) [9]. Accurate TMB analysis relies on expensive genomic platforms, which restricts its feasibility of widespread application. PD-L1 detected by immunohistochemistry (IHC) is unstable due to the anti-PD-L1 antibodies applied in the experiment and the criteria used to determine its positivity. Moreover, PD-L1 expression is characterized by intratumoral [10] and intertumoral heterogeneity, particularly between primary and metastatic biopsies [11, 12], complicating its reliability as a biomarker. Thus, it is still of great value to identify reliable and precise biomarkers with acceptable technical feasibility.

The interaction between oncogenic driver gene alterations and the tumor immune microenvironment influences ICI therapy response [13]. Studies have reported that *EGFR*, *ERBB2 (HER2)*, *STK11* mutations, and *ALK*, *ROS1* and *RET* fusions are associated with reduced ICI response efficacy [12–16]. In contrast, *TP53*, *KRAS*, *BRAF V600E*, *NOTCH4*, *ZFHX3*, *EPHA7*, *SETD2*, *POLE*, and *POLD* mutations are related to superior efficacy [12, 17–20]. In addition, co-occurring genomic alterations contribute to the heterogeneity of the NSCLC microenvironment and make it more complicated to predict ICI response [16, 21]. The concept of variant allele frequency (VAF), defined as the percentage of variant reads divided by the total reads at that locus, has emerged as a noteworthy biomarker [22–24]. In lung adenocarcinoma, low *TP53* VAF is a significant indicator of better anti-PD-(L)1 monotherapy outcomes [25]. However, the potential of a composite mutational VAF profile as a predictive tool in NSCLC ICI therapy warrants further investigation.

In this study, we employed genomic data of 915 NSCLC patients from the database, along with 23 patients from a local dataset, to construct a comprehensive mutational VAF profile that integrates various genetic alterations for personalized treatment strategies. Additionally, we conducted an in-depth analysis of genomic and transcriptomic data of 952 NSCLC patients to explore the underlying mechanism of response to immunotherapy.

## Materials and methods

### Patient data collection and preparation

Clinical information and genomic data, including 781 Memorial Sloan Kettering-Integrated Mutation Profiling of Actionable Cancer Targets (MSK-IMPACT) Sequencing [26] data and 134 whole-exome sequencing (WES) data, were collected from the cBioPortal for Cancer Genomics (https://www.cbioportal.org). Clinical information and tissue samples of the local cohort (n = 23) were collected from the National Cancer Center/National Clinical Research Center for Cancer/Cancer Hospital. MSK patients with progression-free survival (PFS) information were included in MSK cohort 1 (n = 446). And the remaining MSK data with overall survival (OS) information was included in MSK cohort 2 (n = 335). MSK cohort 1 was then randomly divided into a training cohort (70%, n = 313) and a test cohort 1 (30%, n = 133) in a ratio of 7:3. A total of 157 patients with WES data, including Rizvi’s cohort [8] (n = 34), Hellmann’s cohort [27] (n = 75), Miao’s cohort [28] (n = 25), and local cohort (n = 23) were used as test cohort 2. MSK cohort 2 served as test cohort 3. Clinical information, genomic data, and transcriptomic data of 952 NSCLC patients were collected from The Cancer Genome Atlas (TCGA) database (https://portal.gdc.cancer.gov/). Information on the datasets included in the study is shown in Table S1.

Clinical parameters such as age, sex, histological type, smoking status, therapy, and survival information were extracted. The clinical information of the local cohort was collected from electronic medical records. According to the Response Evaluation Criteria in Solid Tumors (RECIST) version 1.1 for NSCLC, clinical response was evaluated using complete response (CR), partial response (PR), stable disease (SD), and progressive disease (PD). To identify the patients with durable clinical benefit (DCB) (PFS > 6 months), we defined those with CR/PR or SD lasting ≥ 6 months as DCB group. Those who experienced PD on or before 6 months were defined as no durable benefit (NDB) group. For the assessment of response, we further evaluated best overall response (BOR), which is an indicator of objective tumor shrinkage and a clearer signal of immunotherapy activity. Specifically, BOR categories include CR/PR and SD/PD. This study was approved by the Medical Ethics Committee of the National Cancer Center/National Clinical Research Center for Cancer/Cancer Hospital.

### Whole-exome sequencing (WES)

After genomic DNA was extracted from the tissue, its quality was verified. Subsequently, qualified genomic DNA was used for library preparation. The DNA libraries were sequenced on the Illumina platform, with raw data recorded in FASTQ format. After data quality control, the sequencing data were mapped to the reference genome (GRCh38). SAMtools mpileup and bcftools were used for variant calling and identifying single nucleotide polymorphisms (SNPs). Detailed steps are shown in the supplementary materials.

### Identification of potential signatures

All the gene mutations included in this study were somatic. The genetic mutation profiles of the patients were extracted from the genomic data, and mutations with a frequency under 5% were eliminated. The Chi-square test (Monte Carlo simulation) was then used to select differential mutational genes between DCB and NDB groups. An adjusted p-value < 0.05 was considered significant.

### Construction and validation of the VAF-related model

The VAF of mutational genes was defined as the percentage of variant reads divided by the total reads at that locus. If a patient had more than one mutational site in a certain gene, the VAF of the gene was calculated as the average of all mutational sites' VAFs.

The random forest algorithm was applied to establish the prediction model using the selected genes, employing the R package “randomForestSRC” (https://cran.r-project.org/web/packages/randomForestSRC/index.html). The training cohort was used to construct the model. The model was then validated in the internal test cohort (test cohort 1) and the external test cohort (test cohort 2), respectively. Besides, test cohort 3 was used to verify the ability of the model in terms of OS.

To estimate the efficacy of the model score, we used R package “pROC” (https://cran.r-project.org/web/packages/pROC/index.html) to calculate the area under the receiver operating characteristic (ROC) curve (AUC). Based on the median of the training cohort, the NSCLC patients were divided into high- and low-score groups. The R package “survminer” (https://cran.r-project.org/web/packages/survminer/index.html) was used to develop Kaplan–Meier (K-M) survival curve and calculate p-values. Moreover, multivariate Cox regression analysis was performed to determine whether the model was independent of age, sex, histological type, therapy, TMB, and PD-L1 expression. The R package “survival” (https://cran.r-project.org/web/packages/survival/index.html) was applied to calculate the hazard ratio (HR), 95% confidence interval (CI), and p-value.

### Genomics analyses

The R package “maftools” [29] (https://www.bioconductor.org/packages/release/bioc/html/maftools.html) was used for mutational analyses and visualization, including oncoplot, transition and transversions (TiTv) analysis, somatic interactions analysis, VAF visualization, and oncogenic pathways enrichment. According to the six types of single nucleotide variant (SNV) and the base types variation upstream and downstream of the SNV position, there are 96 SNV types. In single-base substitution (SBS) analysis, the optimal cluster number was defined according to cophenetic metric of non-negative matrix factorization (NMF) clustering analysis. After comparing the extracted SNV signatures with the Catalogue of Somatic Mutations in Cancer (COSMIC) (https://cancer.sanger.ac.uk/signatures/signaturesv2/) [30] database and calculating the cosine similarity, the SNV signatures were assigned to the most probable SBS signatures. SBS analysis was performed using the R packages “maftools”, “pheatmap”, and “NMF” [31].

### Transcriptomic data preparation and differential analysis

A cohort of 952 NSCLC patients with both genomic and transcriptomic data was identified from the TCGA database. These patients were scored with the constructed VAF-related model and stratified into high- and low-score groups. Gene counts were normalized to fragments per kilobase of transcript per million mapped reads (FPKM). Subsequently, the R package “limma (3.58.1)” (https://www.bioconductor.org/packages/release/bioc/html/limma.html) was utilized to eliminate batch effects when combining datasets. The Wilcoxon rank sum test was utilized for differential analysis between high- and low-score groups and to calculate fold change (FC) values for further mechanism analysis.

### Pathway enrichment analysis

The Weighted Gene Co-expression Network Analysis (WGCNA) employed the R package “WGCNA” [32] (https://cran.r-project.org/web/packages/WGCNA/index.html) to construct the weighted gene co-expression network and select related modules. The top 5,000 genes with the most variation based on median absolute deviation (MAD) were involved in the analysis. The soft threshold parameters were determined when the scale-free topology model fit reached 0.9. An adjacency matrix and a topological overlap matrix (TOM) were then constructed. The minimum number of genes in a module was set to 50. Using the corresponding dissimilarity (1-TOM), hierarchical clustering was performed and modules with a high degree of topological overlap were selected. Pearson’s correlation method was used to find modules relevant to traits, and those strongly correlated with the VAF-related model were selected.

The R package “clusterProfiler” [33] (https://www.bioconductor.org/packages/release/bioc/html/clusterProfiler.html) was used for Gene Ontology (GO) and Kyoto Encyclopedia of Genes and Genomes (KEGG) functional enrichment analysis. The adjusted p-value was calculated using the Benjamini and Hochberg false discovery rate (FDR) method. Both the p-value and FDR < 0.05 were considered significant.

The R package “clusterProfiler” (V3.4.6) was employed for Gene Set Enrichment Analysis (GSEA) with gene sets (c2.cp.kegg.v7.0.symbols.gmt, c5.go.bp.v2023.1.Hs.entrez.gmt) as the background. The normalized enrichment score (NES), nominal p-value, and FDR were calculated to indicate the enrichment and significance of the associated pathways with 1,000 gene set permutations. The FDR-value for significant enrichment pathways was set as 0.05.

### Tumor immune microenvironment (TME) analysis

The estimation of the total immune infiltrate and immune cell subsets in each sample was conducted using CIBERSORTx (https://cibersortx.stanford.edu/) with the LM22 gene set. Additionally, EcoTyper (https://ecotyper.stanford.edu/) was utilized to identify the fundamental cell states and cellular ecosystems constituting NSCLC from bulk transcriptome data [34].

### Immunofluorescence (IF)

The Tyramide Signal Amplification (TSA) technique was used for chromogenic immunostaining. Paraffin sections were backed in a 60 ℃ incubator for 120 min. After dewaxing, hydration, and antigen retrieval, the tissue sections were incubated in blocking buffer to block non-specific binding proteins and endogenous peroxidase activity. Then, the sections were incubated with primary antibody CD8 (diluted at 1:100, #85,336, Cell Signaling Technology) at 37 ℃ for 1 h. After washing, the sections were incubated with a secondary antibody and stained with fluorescein-tyramide (AXT37100031, AlphaTSA Multiplex IHC Kit, Beijing, China) to amplify the signal. The slides were then counterstained with DAPI.

### Statistical analysis

All statistical analyses in this study were performed using GraphPad Prism (version 9.0) and R version 4.2.3 software (https://www.r-project.org/). Categorical variables were described by counts and percentages. Continuous variables were tested for normality before analysis. Normally distributed data were described by mean ± standard deviation, while non-normally distributed data were described by median and quartile. Intergroup comparisons were analyzed using the Chi-square test for categorical variables. Non-normally distributed and normally distributed continuous variables were analyzed using the Mann-Whitey U test and t-test, respectively. The Pearson correlation method was employed in correlation analyses. P-values in K-M analysis were calculated by Log-rank test. A p-value < 0.05 was regarded as statistically significant.

## Results

### Patient characteristics

A total of 915 NSCLC patients from The cBioPortal for Cancer Genomics and 23 NSCLC patients from the local cohort were involved in model construction, model evaluation, and genomics analyses. All 938 patients were treated with anti-PD-(L)1 therapy, including monotherapy, anti-PD-(L)1 in combination with anti-CTLA-4, and anti-PD-(L)1 in combination with chemotherapy. Additionally, 952 NSCLC patients from TCGA were involved in WGCNA, functional enrichment analysis, and tumor immune microenvironment analysis. The flowchart of this study is shown in Figure S1. The information of the included datasets is shown in Table S1. The demographics and clinical characterization of the cohorts are shown in Table S2.

### Construction and validation of the VAF-related model

**Fig. 1:**
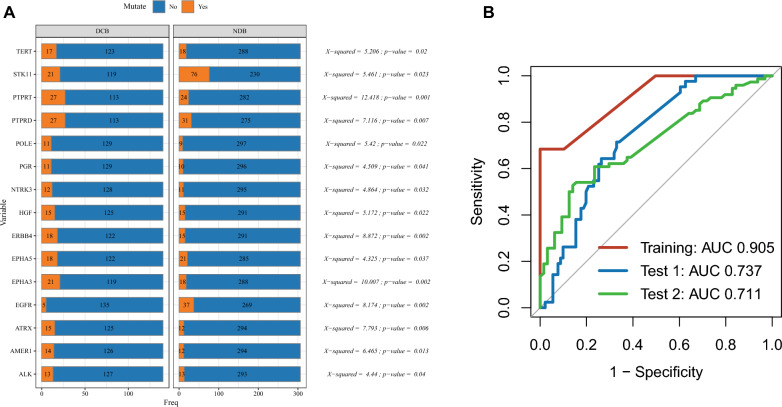
Construction and validation of the variant allele frequency (VAF)-related prediction model. **A** Differential mutate genes between durable clinical benefit (DCB) group and no durable benefit (NDB) group. Chi-square test; A p-value < 0.05 was considered statistically significant. **B** Receiver operating characteristic (ROC) curve of the VAF-related stratification model in the Training, Test 1, and Test 2 cohorts

The genetic mutation profiles of the patients were extracted from genomic data, and mutations with a frequency below 5% were excluded. Subsequently, differentially mutated genes between the DCB and NDB groups were selected using the Chi-squared test. A p-value less than 0.05 was considered statistically significant. A total of 15 genes were identified, including *PTPRT* (***P*** = 0.001), *EGFR* (***P*** = 0.002), *EPHA3* (***P*** = 0.002), *ERBB4* (***P*** = 0.002), and others listed in Fig. 1A and Table S3. We then used the random forest algorithm to construct the model based on the VAF of the selected genes. The ROC-AUC values reached 0.905, 0.737, and 0.711 in the Training (n = 313), Test-1 (internal validation, n = 133), and Test-2 (external validation, n = 157) cohorts, respectively (Fig. 1B).

### High-score group of the VAF-related stratification correlated with better ICI efficacy and favorable prognosis

**Fig. 2:**
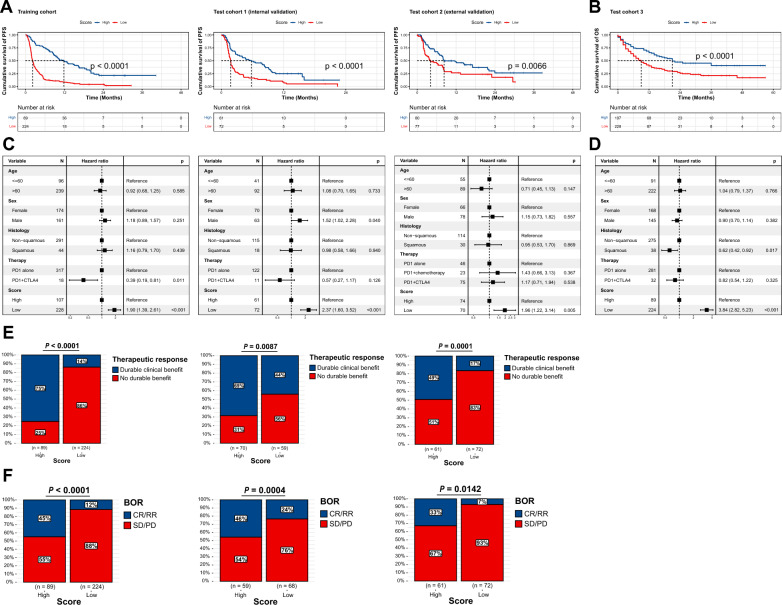
Variant allele frequency (VAF)-related stratification model in predicting immune checkpoint inhibitors (ICIs) response and prognosis. **A–B** Kaplan–Meier (K-M) survival curve of progression-free survival (PFS) in Training, Test 1, Test 2 cohorts, and overall survival (OS) in Test 3 cohort. The p-value was calculated using the log-rank test. **C–D** Multivariate Cox regression analysis of risk score level and clinical characteristics in the Training, Test 1, Test 2, and Test 3 cohorts. **E** Proportion of durable clinical benefit (DCB) and no durable benefit (NDB) patients in high- and low-score groups. Chi-square test; A p-value < 0.05 was considered statistically significant. **F** Proportion of complete response (CR)/partial response (PR) and stable disease (SD)/progressive disease (PD) patients in high- and low-score groups. Chi-square test; A p-value < 0.05 was considered statistically significant

All the patients were divided into high- and low-score groups according to the same threshold, which was the median of the training cohort (threshold = 0.2645). The K-M survival curve of the high-score group presented a preferable median PFS (mPFS) (Training: 9.70 months vs. 2.20 months, ***P*** < 0.0001; Test-1: 5.77 months vs. 1.80 months, ***P*** < 0.0001; Test-2: 7.61 months vs. 3.65 months, ***P*** = 0.0066) (Fig. 2A) and OS (Test-3 [n = 341]: 15.00 months vs. 8.00 months, ***P*** < 0.0001) (Fig. 2B), compared with the low-score group. We noticed that clinical characteristics, such as therapy types, may affect outcomes (Figure S2). To eliminate the influence of confounding factors, we included clinical parameters in a multivariate Cox regression model. After correction, the stratification system remained statistically significant in predicting PFS (Fig. 2C) and OS (Fig. 2D), making the results more robust and reliable. The high-score group also contained a higher proportion of DCB patients (Training: ***P*** < 0.0001, Test-1: ***P*** = 0.0087, Test-2: ***P*** = 0.0001). And for the assessment of BOR, the high-score group contained a higher proportion of CR/PR patients (Training: ***P*** < 0.0001, Test-1: ***P*** = 0.0004, Test-2: ***P*** = 0.0142), indicating a favorable response to ICIs (Fig. 2E and 2F).

### VAF-related stratification combined with PD-L1 or TMB can efficiently differentiate ICI response

**Fig. 3:**
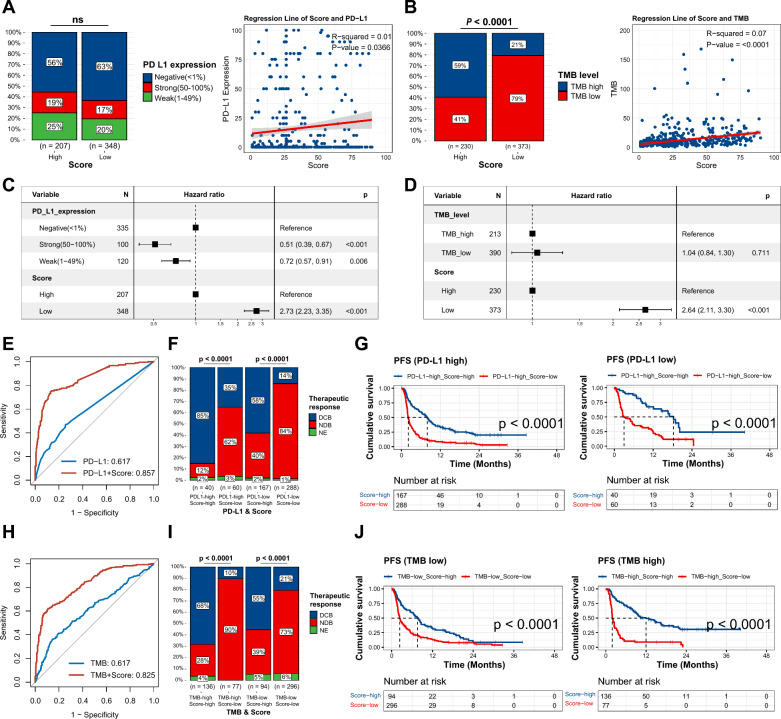
Variant allele frequency (VAF)-related stratification model in combination with programmed death-ligand 1 (PD-L1) or tumor mutation burden (TMB). **A–B** Comparison of PD-L1 and TMB between high- and low-score groups, and correlation analysis of model score with PD-L1 expression or TMB. PD-L1 expression was classified as negative (< 1%), weak (1–49%), and strong (50–100%). TMB was categorized as low (< 10 mut/Mb) and high (≥ 10 mut/Mb). Chi-square test and Pearson correlation analysis were conducted. **C–D** Multivariate Cox regression analysis of PD-L1 or TMB and the model. **E** Receiver operating characteristic (ROC) curve of PD-L1 alone and in combination with the model. **F** Proportion of durable clinical benefit (DCB) and no durable benefit (NDB) patients between high- and low-score groups in PD-L1 high and PD-L1 low groups, respectively. PD-L1 expression < 1% was considered PD-L1 low, while 1–100% was considered PD-L1 high. Chi-square test. **G** Kaplan–Meier (K-M) survival curve of progression-free survival (PFS) between high- and low-score groups in PD-L1 high and PD-L1 low groups, respectively. The p-value was calculated from log-rank test. **H** ROC curve of TMB alone and in combination with the model. **I** Proportion of DCB and NDB patients between high- and low-score groups in TMB-high and TMB-low groups, respectively. Chi-square test. **J** K-M survival curve of PFS between high- and low-score groups in TMB-high and TMB-low groups, respectively. The p-value was calculated from log-rank test

Subsequently, we investigated the association between the VAF-related stratification model and known biomarkers for ICIs, namely TMB and PD-L1. We found no significant correlation between PD-L1 and the stratification system but a weak correlation with the model score (r^2^ = 0.01, ***P*** = 0.0366) (Fig. 3A). In contrast, a higher TMB was linked to the high-score category (***P*** < 0.0001), and the model score showed a significant correlation with TMB (r^2^ = 0.07, ***P*** < 0.0001) (Fig. 3B). To verify the independence of the model from PD-L1 and TMB, we integrated the stratification model into a multivariate Cox regression analysis. After adjustment, the VAF-related stratification remained a statistically significant prognostic factor (Fig. 3C and 3D). This indicates that the model may influence prognosis through mechanisms independent of PD-L1 and TMB.

We then assessed the efficacy of TMB and PD-L1 in conjunction with our model. The ROC-AUC of PD-L1 improved from 0.617 to 0.857 when combined with the model (Fig. 3E). Similarly, the performance of TMB increased from 0.617 to 0.825 (Fig. 3H). For the purpose of subgroup comparisons, we defined PD-L1 expression levels below 1% as ‘PD-L1 low’ and levels between 1–100% as ‘PD-L1 high’. A TMB of less than 10 mutations per megabase (mut/Mb) was categorized as ‘TMB-low’, while a TMB of 10 mut/Mb or above was considered ‘TMB-high’. Notably, a substantially greater proportion of patients who experienced DCB were found in the high-score group, regardless of whether they had high (***P*** < 0.0001) or low (***P*** < 0.0001) PD-L1 expression (Fig. 3F). When PD-L1 was assessed in conjunction with the VAF-related model, the two factors together provided a more distinct PFS differentiation for both high (***P*** < 0.0001) and low (***P*** < 0.0001) PD-L1 groups (Fig. 3G). The high-score group also manifested a higher proportion of DCB in both the TMB-high (***P*** < 0.0001) and TMB-low (***P*** < 0.0001) populations (Fig. 3I). Furthermore, when TMB was considered alongside our stratification model, the combined factors significantly improved the distinction of the PFS curves for both TMB-high (***P*** < 0.0001) and TMB-low (***P*** < 0.0001) groups (Fig. 3J). This combination addressed the limitations of using PD-L1 and TMB alone (Figure S3).

Collectively, these results suggest that the mutational VAF profile is a potent adjunctive biomarker that holds promise for increasing the accuracy of PD-L1 expression and TMB in differentiating therapeutic responses to ICIs.

### Mutational landscape profiling of VAF-related stratification system

**Fig. 4:**
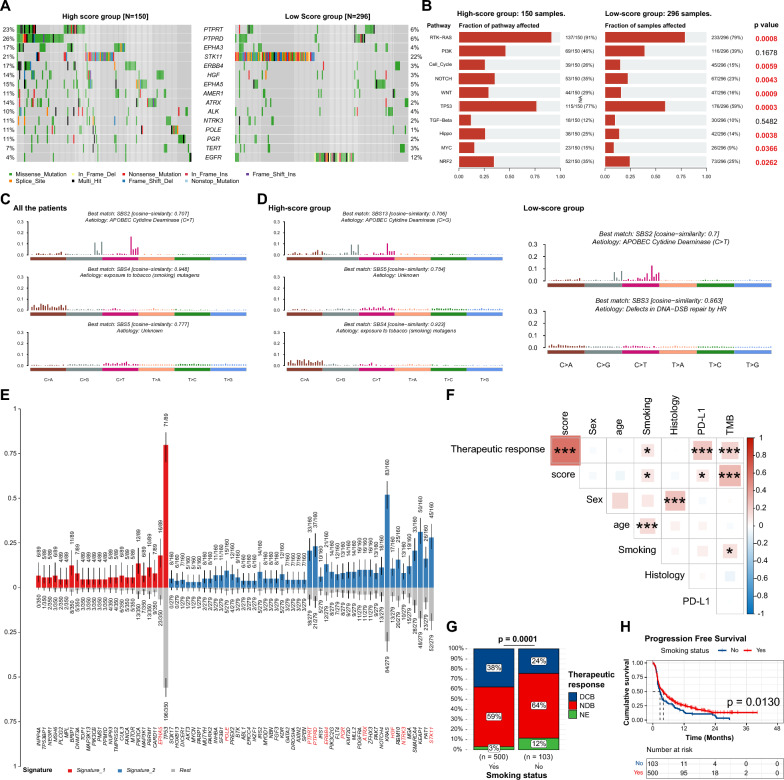
Mutational landscape profiling of VAF-related stratification system **A** Waterfall plot of the 15 genes involved in the model. **B** Oncogenic pathways enrichment analysis. **C** Signature analysis in all patients. **D** Signature analysis in high-score and low-score groups, respectively. **E** Characteristic genes of the signatures. Red bars are the characteristic genes in signature 1. Blue bars are the characteristic genes in signature 2. The red genes below are involved in the VAF-related stratification model. A p-value < 0.05 was considered statistically significant. **F** Correlation analysis of model score with clinical parameters using Pearson correlation analysis. Levels of significance: *: P < 0.05; **: P < 0.01; ***: P < 0.001. **G** Proportion of durable clinical benefit (DCB) and no durable benefit (NDB) patients between smoking and non-smoking groups. Chi-square test. **H** Comparison of Kaplan-Meier **K**–**M** survival curve of progression-free survival (PFS) between smoking and non-smoking groups. The p-value was calculated from log-rank test.

The basic mutational analyses of the cohorts were presented in Figure S4-5. To validate the mutational profile of the 15 genes involved in the model, we drew an oncoplot of these genes, delineating distinctions between the high- and low-score groups. We observed that *EGFR* and *STK11* alterations were predominantly enriched in the low-score group. Whereas alterations in the remaining 13 genes were more frequent within the high-score group (Fig. 4A). Additionally, the high-score group exhibited a higher fraction of patients involved in oncogenic signaling pathways alterations. This finding was statistically significant in the RTK-RAS pathway (***P*** = 0.0008), cell cycle pathway (***P*** = 0.0059), NOTCH pathway (***P*** = 0.0043), WNT pathway (***P*** = 0.0009), TP53 pathway (***P*** = 0.0003), Hippo pathway (***P*** = 0.0038), MYC pathway (***P*** = 0.0366), and NRF2 pathway (***P*** = 0.0262) (Fig. 4B), suggesting a potential biological underpinning for risk categorization. The complete gene profiles of the pathways were shown in Figure S6.

SBS signatures were discerned to reveal underlying mutagenic processes. SBS2 (APOBEC Cytidine Deaminase [C > T]), SBS4 (exposure to tobacco [smoking] mutagens), and SBS5 (Unknown) demonstrated variations in distribution between the two groups (Figure S7 and Fig. 4C). Particularly, Signature 2 (SBS4), which is associated with a smoking history, was significantly more prevalent in the high-score group. In contrast, signature 1 (SBS2, APOBEC Cytidine Deaminase [C > T]) was more abundant in the low-score group (Fig. 4D). Moreover, of the 13 genes enriched in the high-score group, nine were also characteristic of Signature 2 (the red genes under the blue bars) (Fig. 4E). These results illustrated the strong association between the high-score group and smoking. Further correlation analysis demonstrated a direct association between smoking, ICIs response, and the VAF stratification system (Fig. 4F). The smoking population tended to achieve a higher proportion of DCB (Fig. 4G), and K-M analysis showed a significantly improved PFS in smoking patients (Fig. 4H).

Separate analyses of SBS signatures within the high- and low-score groups provided further insights. In the high-score group, signatures corresponded to SBS13 (APOBEC Cytidine Deaminase [C > G]), SBS5 (Unknown), and SBS4 (exposure to tobacco [smoking] mutagens), respectively. Signatures extracted from the low-score group matched SBS2 (APOBEC Cytidine Deaminase [C > T]) and SBS3 (defects in DNA-double-strand break [DSB] repair by homologous recombination [HR]) (Figure S7 and Fig. 4D). These divergent profiles suggest that various etiological factors may underlie the distinct mutational landscapes, potentially influencing the therapeutic responses to ICIs.

### Transcriptomic landscape mapping of the dysregulated pathways in VAF-related stratification system

**Fig. 5:**
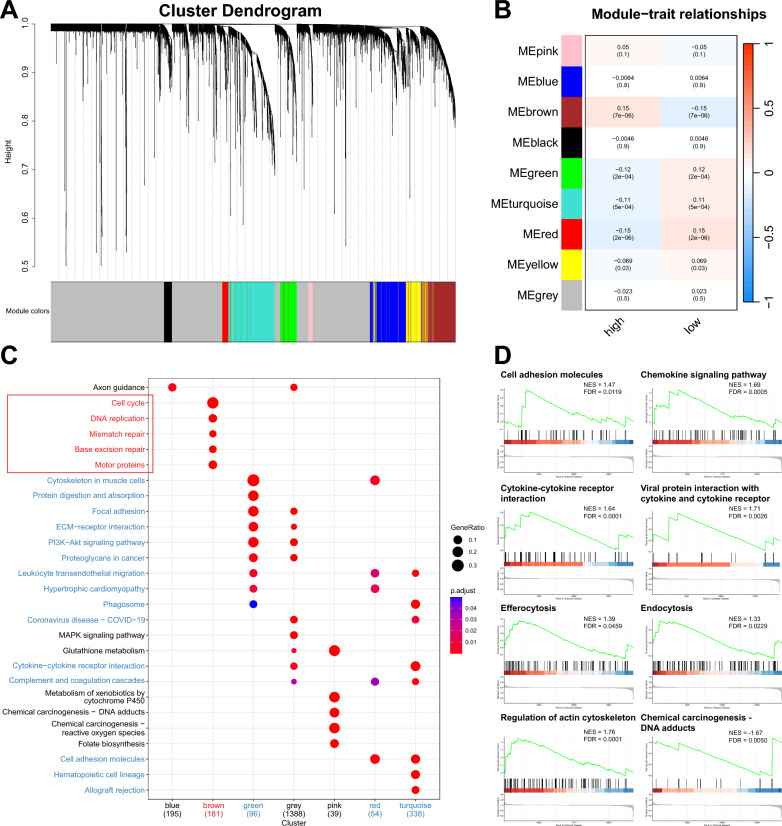
Transcriptomic landscape mapping of the dysregulated pathways in VAF-related stratification system **A** Clustering dendrogram obtained by average linkage hierarchical clustering. The color row underneath the dendrogram shows the module assignment determined by the Dynamic Tree Cut. **B** Module-trait relationships. Each row corresponds to one module eigengene (labeled by color). Blue color represents a negative correlation, while red represents a positive correlation. Pearson correlation coefficient (p-value). **C** Kyoto encyclopedia of genes and genomes (KEGG) module enrichment analysis. Red modules and pathways are positively related to the high-score group. Blue modules and pathways are negatively related to the low-score group. Fisher's exact test. The adjusted p-value was calculated by Benjamini and Hochberg false discovery rate (FDR) method. FDR < 0.05 was considered statistically significant. The plot showed the top 5 pathways of each module. **D** Pathways enriched in the high- and low-score group in Gene set enrichment analysis (GSEA) of the whole gene set. NES, normalized enrichment risk. NES > 0 indicated the pathway was enriched in the low-score group. NES < 0 indicated the pathway was enriched in the high-score group. FDR, false discovery rate. FDR < 0.05 was considered statistically significant.

To elucidate the biological underpinnings of the model, we conducted a comprehensive analysis of the genomic and transcriptomic data from 952 NSCLC patients derived from TCGA. Each patient was evaluated using the VAF-related model. Subsequently, WGCNA identified 5 modules correlated with the stratification system. Four of these modules—green, turquoise, red, and yellow—were associated with the low-score group, while the brown module was indicative of the high-score group (Fig. 5A-B, Figure S8 and Table S4).

Functional pathway analysis of the characteristic genes within these modules was performed using GO and KEGG enrichment analyses (Table S8-9). The low-score gene modules were notably enriched in pathways pertaining to cellular interaction, inflammation, and cytoskeleton organization, such as cytoskeleton in muscle cells, ECM-receptor interaction, focal adhesion, leukocyte transendothelial migration, cytokine-cytokine receptor interaction, and complement and coagulation cascades. Conversely, the high-score group exhibited significant enrichment in DNA damage response (DDR) pathways, such as cell cycle, DNA replication, base excision repair (BER), mismatch repair (MMR), and homologous recombination (HRR) (Fig. 5C and Figure S9-10).

GSEA of the whole gene set further substantiated these findings. The analysis demonstrated enrichment for cell adhesion molecules, chemokine signaling pathway, cytokine-cytokine receptor interaction, viral protein interaction with cytokine and cytokine receptor, efferocytosis, endocytosis, and regulation of actin cytoskeleton in the low-score group (Fig. 5D). In the high-score group, GSEA revealed enrichment for chemical carcinogenesis-DNA adducts pathway (Fig. 5D).

### Reshape of tumor immune microenvironment in VAF-related stratification system

**Fig. 6:**
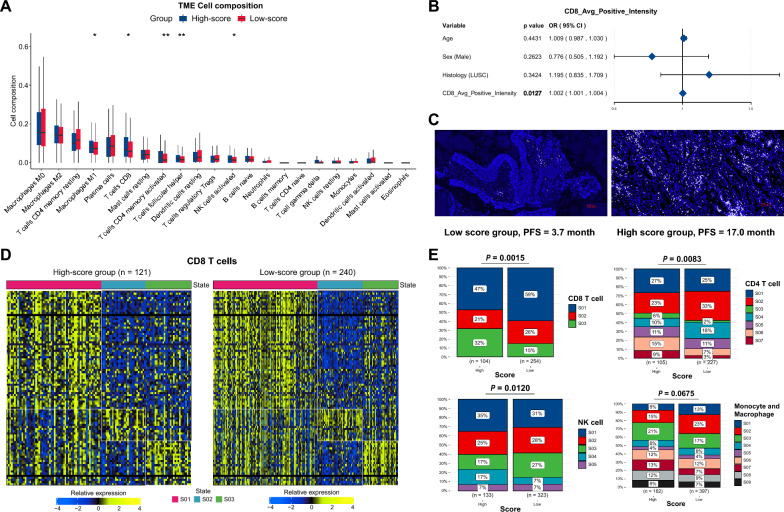
Tumor immune microenvironment analysis in VAF-related stratification system **A** Comparison of infiltrated immune cells between the high- and low-score groups. The P value was calculated by Wilcoxon rank sum test. **B** Multivariate logistic regression model of the clinical factors and CD8 average positive intensity of immunofluorescence (IF) staining in local cohort. P values calculated from multivariable logistic regression. **C** IF staining of CD8 average positive intensity in patients of low- and high-score group, respectively. The color “white” indicated CD8 staining, the color “blue” indicated DAPI for nuclear visualization. PFS, progression-free survival. Average CD8 positive intensity, high-score group (DCB, PFS = 17.0 month): 331.87, low-score group (NDB, PFS = 3.7 month): 215.79. **D** Heatmap of CD8 T cell states. Cell states: Naive/central memory (S01), Late-stage differentiated effector (S02), Exhausted/effector memory (S03). **E** Cell states of CD8 T cell, CD4 T cell, NK cell, and Monocyte/Macrophage between high- and low-score groups. Chi-square test. The detailed cell states of each cell type were shown in Table S7. Levels of significance: *: P < 0.05; **: P < 0.01; ***: P < 0.001; ****: P < 0.0001.

To investigate TME discrepancies between the high- and low-score groups, we employed the CIBERSORTx algorithm to calculate the composition of tumor-infiltrating immune cells. The high-score group exhibited a higher abundance of immune cells. We identified five cell types with significant differences between the two groups. The low-score group contained a reduced proportion of CD8^+^T cells (***P*** = 0.0236), activated memory CD4^+^T cells (***P*** = 0.0079), follicular helper T cells (Tfh) (***P*** = 0.0066), activated NK cells (***P*** = 0.0322), and M1 macrophage (***P*** = 0.0117) (Fig. 6A and Table S5). Additionally, IF staining was performed to assess CD8^+^T cell presence within the local cohort of 23 patients. Logistic regression analysis showed that the CD8^+^T cells was statistically associated with the high-score group (***P*** = 0.0127) (Fig. 6B). Figure 6C illustrates a comparison of staining intensity in a typical case of the high-score group (PFS = 17.0 months, average CD8 positive intensity: 331.87) and a case of the low-score group (PFS = 3.7 months, average CD8 positive intensity: 215.79).

Moreover, we applied Ecotyper [34] to further estimate the detailed cell states of each cell type. We found that exhausted/effector memory (S03) CD8 T cells were significantly elevated in the high-score group (***P*** = 0.0015), which may account for the preferable response to ICIs in this group (Fig. 6D–E). Classical M1 (S03), which demonstrate an anti-tumor function, was also higher in the high-score group, while Classical M0 (normal-enriched) (S02) was lower (***P*** = 0.0675) (Fig. 6E). This result suggests a potential transformation from M0 to M1 in the high-score group. CD4 T cell (***P*** = 0.0083) and NK cell (***P*** = 0.0120) also showed statistically different compositions of cell states (Fig. 6E). Other cell types were shown in the supplementary materials (Figure S11 and Table S6–7).

In summary, our findings highlight the critical role of gene alterations in reconfiguring the TME, which consequently influences tumor immunity and modulates responses to ICI therapies. The pronounced disparity in CD8^+^T cell infiltration between the high- and low-score groups particularly illustrates the immune mediation role of genomic variations in ICI treatment.

## Discussion

Despite extensive efforts being made in the field of immunotherapy [6–8], the availability of predictive biomarkers for ICIs remains limited, and the mechanisms underlying ICI resistance are not fully understood. Gene alterations have shown prominent contributions in reshaping TME and influencing responses to ICIs [13–16]. The mutational VAF may serve as a promising and reliable biomarker. To address the shortcomings of existing biomarkers, we focused on oncogenetic mutational VAF and developed a stratification system aimed at differentiating ICI responses in patients with NSCLC. Subsequently, we investigated the mutational and transcriptomic landscape associated with the VAF-related model and analyzed the composition of tumor-infiltrating immune cells in high- and low-score groups.

The MSK cohort was utilized in model construction and internal validation, leveraging MSK-IMPACT Sequencing—a hybridization capture-based next generation sequencing (NGS) assay. An external cohort employing WES assay further validated the model. The consistency across these disparate platforms bolsters the robustness of the VAF-related model. Additionally, VAF detection is potentially a more stable, technically feasible, and cost-effective predictor compared to current biomarkers. Recent studies have demonstrated the ability to detect VAFs as low as 0.1% using approaches such as low-depth NGS or quantitative PCR with multiplex blocker displacement amplification (mBDA) method [35, 36], further supporting VAF's utility as a clinical biomarker.

The majority of gene alterations implicated in our model, including *PTPRT*, *EPHA3*, *ERBB4*, *ATRX*, *PTPRD*, *AMER1*, *TERT*, *HGF*, *POLE*, *NTRK3*, *EPHA5*, *ALK*, and *PGR*, were positively correlated with the high-score group and favorable ICI responses. For some of these genes, like *PTPRT* [37, 38], *PTPRD* [38], *ERBB4* [39], *ATRX* [40], *TERT* [41], *POLE* [8], *NTRK3* [42], *EPHA5* [43], and *ALK* [14], previous literature has highlighted their potential roles as biomarkers for ICIs therapy in NSCLC. Our study firstly reported the significance of *EPHA3*, *AMER1*, *HGF*, and *PGR* alterations. Consistent with prior research, these genes were associated with DDR pathways, a higher TMB, and increased infiltration of tumor infiltrating lymphocytes (TILs). TERT was a catalytic subunit of telomerase, and played an important role in cancer proliferation, invasion, and DNA damage response [44, 45]. *POLE* encoded the catalytic and proofreading subunits of DNA polymerase, affecting DNA replication and proofreading [46]. EPHA3 was reported related to tumor-specific antigens presented by HLA class II molecules to CD4^+^T cells [47]. HGF was positively correlated with PD-L1 and promoted immune escape in EGFR-TKI resistance NSCLC patients [48]. *PTPRT* mutations were related to a higher TMB, and increased infiltration of TILs [37]. *PTPRD/PTPRT* co-mutations exhibited an improved immune-activated phenotype than mutated alone [38]. *ERBB4*, *NTRK3*, and *EPHA5* mutations also had similar effects on TMB and TILs [39, 42, 43].

Interestingly, gene alterations in *EGFR* and *STK11* attracted an immunosuppressive TME. Our findings revealed a decrease in TILs, especially a decrease in exhausted/effector memory CD8 T cells, in the low-score group, characterized by an accumulation of *EGFR* and *STK11* mutations. Previous research has shown that *EGFR* mutations can lead to reduced PD-L1 expression, diminished T-cell infiltration, and a shrinking proportion of PD-L1^+^/CD8^+^ TILs [49]. A lack of CD8^+^ tissue-resident memory (TRM) cells in *EGFR*-mutated lung adenocarcinoma might be a key factor contributing to a suppressive TME [50]. Tumors with *STK11* mutations are typified by excessive production of pro-inflammatory cytokines (IL6, G-CSF, and CXCL-7), accumulation of neutrophils with T-cell suppressive capacities, and low expression of PD-L1[16, 51]. Hence, specific gene alterations can remodel the TME, resulting in diverse responses to ICIs. However, the intrinsic regulatory mechanisms linking gene alterations and the TME represent a complex and dynamic system. DDR-related mutations, for example, can increase genomic instability, generate tumor neoantigens, and improve tumor recognition by the adaptive immune system, leading to TIL recruitment [52, 53]. Conversely, certain oncogene mutations can attract immunosuppressive, resulting in a CD8^+^T cell-deficient TME.

Our mutational signature analysis highlighted significant differences between high- and low-score groups. In regard to APOBEC-driven SBS signatures, SBS2 (APOBEC C > T) was enriched in the low-score group, while SBS13 (APOBEC C > G) was enriched in the high-score group. The high-score group was also characterized by SBS4 (exposure to tobacco [smoking] mutagens). A recent genomic study revealed that tobacco-driven SBS4 and APOBEC signature SBS13 were enriched in stop-gain mutations (SGMs) in various cancer types [54]. C > G and C > A mutations are common to SBS13, while C > T mutations are common to SBS2. This accounted for why SBS13 was preferable to SBS2 in converting codons to stop codons (TAG, TAA, and TGA). Notably, SGMs are the most disruptive class of SNVs that induces protein loss of function (LOF), implicating these possibly as sources of neoantigens and correlating with increased TILs in the high-score group.

Due to the complexity of co-mutation and gene interactions, developing a collaborative mutational profile to integrate complementary oncogenes is of critical importance for personalized oncology. In this study, we constructed a VAF-related model and integrated genomic and transcriptomic data to probe the initial mechanisms underlying the response to ICIs. Inevitably, there are several limitations. In clinical practice, combinations of anti-PD-(L)1 therapy with other treatments, such as chemotherapy or anti-angiogenic therapy, are commonly used. The number of patients using combined anti-PD-(L)1 therapies in this study was limited. It is essential to verify the stratification system and investigate resistance mechanisms within a larger cohort receiving various combined ICI therapies. Additionally, our study lacks access to randomized trial data. Ideally, a test for interaction between treatment exposure and the biomarker would be necessary to verify the predictive role of the biomarker [55]. Future research should aim at validating the model in randomized controlled trials to better elucidate its predictive capabilities. Furthermore, the detailed biological functions of the mutated genes within the TME warrant more in-depth investigation. Future work should also focus on developing reliable detection methods with acceptable technical feasibility and attempting to validate the model based on circulating tumor DNA (ctDNA), which will further extend the clinical application of the model.

In conclusion, we have established a VAF-related model for differentiating ICI response in NSCLC, highlighting the importance of precision therapy. The VAF mutational profile provides a valuable biomarker that enhances the stratification and treatment management of NSCLC patients using ICI therapy. Notably, it complements PD-L1 and TMB biomarkers, demonstrating its application in clinical personalized medicine.

### Supplementary Information

Supplementary material 1.

Supplementary material 2.

Supplementary material 3.

Supplementary material 4.

## Data Availability

The data associated with the findings of this study are available in the main text or supplementary materials. The raw data that support this study are available from the corresponding author upon reasonable request.

## Abbreviations

AUC: Area under the curve
BOR: Best overall response
COSMIC: Catalogue of somatic mutations in cancer
CR: Complete response
CI: Confidence interval
CTLA-4: Cytotoxic T-lymphocyte antigen 4
DCB: Durable clinical benefit
FC: Fold change
FPKM: Fragments per kilobase of transcript per million mapped reads
GO: Gene ontology
GSEA: Gene set enrichment analysis
HR: Hazard ratio
ICIs: Immune checkpoint inhibitors
IF: Immunofluorescence
IHC: Immunohistochemistry
K-M: Kaplan–Meier
KEGG: Kyoto Encyclopedia of Genes and Genomes
LASSO: Least Absolute Shrinkage and Selection Operator
LOF: Loss of function
MAD: Median absolute deviation
mPFS: Median progression-free survival
MSK-IMPACT: Memorial Sloan Kettering-Integrated Mutation Profiling of Actionable cancer targets
MSI: Microsatellite instability
dMMR: Mismatch-repair deficiency
mBDA: Multiplex blocker displacement amplification
NGS: Next generation sequencing
NDB: No durable benefit
NMF: Non-negative matrix factorization
NSCLC: Non-small cell lung cancer
NES: Normalized enrichment score
OS: Overall survival
PR: Partial response
PD-1: Programmed death 1
PD-L1: Programmed death-ligand 1
PD: Progressive disease
PFS: Progression-free survival
ROC: Receiver operating characteristic
RECIST: Response evaluation criteria in solid tumors
SBS: Single-base substitution
SNPs: Single nucleotide polymorphisms
SNV: Single nucleotide variant
SD: Stable disease
SGMs: Stop-gain mutations
TCGA: The Cancer Genome Atlas
TOM: Topological overlap matrix
TiTv: Transversions
TME: Tumor immune microenvironment
TILs: Tumor infiltrating lymphocytes
TMB: Tumor mutation burden
TSA: Tyramide signal amplification
VAF: Variant allele frequency
WGCNA: Weighted gene co-expression network analysis
WES: Whole-exome sequencing
