# Supplementary material for: Mutational variant allele frequency profile as a biomarker of response to immune checkpoint blockade in non-small cell lung Cancer
Source: J Transl Med. 2024 Jun 18;22:576. doi: 10.1186/s12967-024-05400-7 (PMC11184775; doi:10.1186/s12967-024-05400-7)
Supplement: Supplementary file 1 — Supplementary material 1. [file 12967_2024_5400_MOESM1_ESM.docx]

**Supplementary Materials**

**Whole exome sequencing (WES)**

1. **Experimental Procedure**
   1. **Sample Quality Control**

After extracted the genomic DNA from tissue, the quality of isolated genomic DNA was verified. DNA degradation and contamination were monitored on 1% agarose gels. DNA concentration was measured by Qubit® DNA Assay Kit in Qubit® 3.0 Flurometer (Invitrogen, USA). DNA concentration ≥20 ng/µL and the total amount of DNA >0.4 µg was considered qualified.

**1.2 Library Preparation**

The exome sequences were efficiently enriched from genomic DNA using Agilent liquid capture system (Agilent SureSelect Human All Exon V6). Firstly, qualified genomic DNA was randomly fragmented to an average size of 180-280bp by Covaris S220 sonicator. Remaining overhangs were converted into blunt ends via exonuclease polymerase activities. Secondly, DNA fragments were end repaired and phosphorylated, followed by A-tailing and ligation at the 3’ends with paired-end adaptors. DNA fragments with ligated adapter molecules on both ends were selectively enriched in a PCR reaction. After PCR reaction, libraries hybridize with liquid phase with biotin labeled probe, then use magnetic beads with streptomycin to capture the exons of genes. Captured libraries were enriched in a PCR reaction to add index tags to prepare for sequencing. Products were purified using AMPure XP system (Beckman Coulter, Beverly, USA), DNA concentration was measured by Qubit®3.0 Flurometer (Invitrogen, USA), libraries were analyzed for size distribution by NGS3K/Caliper and quantified by real-time PCR (3 nM). At last, DNA library were sequenced on Illumina for pairedend 150 bp reads.

**1.3 Clustering & Sequencing**

The clustering of the index-coded samples was performed on a cBot Cluster Generation System using Illumina PE Cluster Kit (Illumina, USA) according to the manufacturer’s instructions. After cluster generation, the DNA libraries were sequenced on Illumina platform and 150 bp paired-end reads were generated.

1. **Bioinformatics Analysis Pipeline**

**2.1 Data Quality Control**

The original fluorescence image files obtained from Illumina platform are transformed to short reads (Raw data) by base calling and these short reads are recorded in FASTQ format, which contains sequence information and corresponding sequencing quality information. To guarantee meaningful downstream analysis, we set strict quality control steps. All the downstream bioinformatics analyses were based on the high quality clean data, which were retained after these steps. The steps of data processing were as follows:

(1) Discard a paired reads if either one read contains adapter contamination (>10 nucleotides aligned to the adapter, allowing ≤ 10% mismatches);

(2) Discard a paired reads if more than 10% of bases are uncertain in either one read;

(3) Discard a paired reads if the proportion of low quality (Phred quality <5) bases is over 50% in either one read.

**2.2 Reads Mapping to Reference Sequence**

Valid sequencing data is mapped to the reference genome (GRCh37/hg19/GRCh38) by BurrowsWheeler Aligner (BWA) software[1] to get the original mapping result in BAM format. Subsequently, Samtools[2] and Sambamba[3] are spectively utilized to sort bam files, do duplicate-marking to generate final bam file. If one or one pair read(s) has multiple mapping positions, the strategy adopted by BWA are to select the best one, if there are multi best mapping position, we randomly pick one. These duplicate reads are uninformative and shouldn’t be considered as evidence for variants. Sambamba is employed to mark these duplicates so that we will ignore them in the following analysis.

**2.3 Variant detection and annotation**

SAMtools[2] mpileup and bcftools were used to do variant calling and identify SNP.

ANNOVAR[4] is performed to do annotation for VCF (Variant Call Format) file obtained in the previous step. The variant position, variant type, conservative prediction and other information are obtained at this step through a variety of databases, such as dbSNP, 1000 Genome, GnomAD, CADD and HGMD. Since we are interested in exonic variants, gene transcript annotation databases, such as Consensus CDS, RefSeq, Ensemble and UCSC, are also applied for annotation to determine amino acid alternation.

[1] H. Li, R. Durbin, Fast and accurate short read alignment with Burrows-Wheeler transform, Bioinformatics, 25 (2009) 1754-1760.

[2] H. Li, B. Handsaker, A. Wysoker, T. Fennell, J. Ruan, N. Homer, G. Marth, G. Abecasis, R. Durbin, S. Genome Project Data Processing, The Sequence Alignment/Map format and SAMtools, Bioinformatics, 25 (2009) 2078-2079.

[3] A. Tarasov, A.J. Vilella, E. Cuppen, I.J. Nijman, P. Prins, Sambamba: fast processing of NGS alignment formats, Bioinformatics, 31 (2015) 2032-2034.

[4] K. Wang, M. Li, H. Hakonarson, ANNOVAR: functional annotation of genetic variants from high-throughput sequencing data, Nucleic Acids Res, 38 (2010) e164.
