# Supplementary material for: Mutational variant allele frequency profile as a biomarker of response to immune checkpoint blockade in non-small cell lung Cancer
Source: J Transl Med. 2024 Jun 18;22:576. doi: 10.1186/s12967-024-05400-7 (PMC11184775; doi:10.1186/s12967-024-05400-7)
Supplement: Supplementary file 2 — Supplementary material 2. [file 12967_2024_5400_MOESM2_ESM.docx]

**Supplementary materials for**

Mutational variant allele frequency profile as a biomarker of response to immune checkpoint blockade in non-small cell lung cancer

**The PDF file includes:**

Tables S1 to S7

Figures S1 to S11

**Table S1.** **The information of the included datasets in the study.**

| **Source** | **Platform** | **Cohort name** | **Included samples** | **Purpose** |
| --- | --- | --- | --- | --- |
| MSK | MSK-IMPACT Sequencing | Training cohort | 313 | Identification of the risk genes and construction of the model |
|  |  | Test cohort 1 | 133 | Identification of the risk genes and internal validation of the model |
| Rizvi’s cohort,  Hellmann’s cohort, Miao’s cohort, and local cohort | WES | Test cohort 2 | 157 | External validation of the model |
| MSK | MSK-IMPACT Sequencing | Test cohort 3 | 341 | Validation in OS prediction |
| TCGA | WES and RNA-seq | TCGA cohort | 952 | Mechanism analysis |
| Local cohort | IF | Local cohort | 23 |  |
| **Abbreviations:** non-small cell lung cancer, NSCLC; Memorial Sloan Kettering Cancer Center, MSK; Memorial Sloan Kettering-Integrated Mutation Profiling of Actionable Cancer Targets, MSK-IMPACT; whole-exome sequencing, WES; overall survival, OS; The Cancer Genome Atlas, TCGA; immunofluorescence, IF. | | | | |

**Table S2. Demographics and clinical characterization of the cohorts**

| **Characteristic** | **Training cohort**  **N=313 (%)** | **Test cohort 1**  **N=133 (%)** | **Test cohort 2**  **N=157 (%)** | **Test cohort 3**  **N=335 (%)** | **TCGA cohort**  **N=952 (%)** | **Local cohort**  **N=23 (%)** |
| --- | --- | --- | --- | --- | --- | --- |
| **Age** |  |  |  |  |  |  |
| <=60 | 91 (29%) | 41 (31%) | 55 (35%) | 96 (29%) | 256 (27%) | 7 (30%) |
| >60 | 222 (71%) | 92 (69%) | 89 (57%) | 239 (71%) | 668 (70%) | 16 (70%) |
| Unknown | 0 (0%) | 0 (0%) | 13 (8%) | 0 (0%) | 28 (3%) | 0 (0%) |
| **Sex** |  |  |  |  |  |  |
| Female | 168 (54%) | 70 (53%) | 75 (48%) | 174 (52%) | 381 (40%) | 3 (13%) |
| Male | 145 (46%) | 63 (47%) | 82 (52%) | 161 (48%) | 571 (60%) | 20 (87%) |
| **Histology** |  |  |  |  |  |  |
| Non-squamous | 275 (88%) | 115 (86%) | 127 (81%) | 291 (87%) | 467 (49%) | 13 (57%) |
| Squamous | 38 (12%) | 18 (14%) | 30 (19%) | 44 (13%) | 485 (51%) | 10 (43%) |
| **Smoking status** |  |  |  |  |  |  |
| Current | 37 (12%) | 17 (13%) | 28 (18%) | 0 (0%) | 0 (0%) | 5 (22%) |
| Former | 229 (73%) | 93 (70%) | 96 (61%) | 0 (0%) | 0 (0%) | 14 (61%) |
| Never | 47 (15%) | 23 (17%) | 33 (21%) | 0 (0%) | 0 (0%) | 4 (17%) |
| Unknown | 0 (0%) | 0 (0%) | 0 (0%) | 335 (100%) | 952 (100%) | 0 (0%) |
| **PD-L1 expression** |  |  |  |  |  |  |
| Negative (<1%) | 215 (69%) | 88 (66%) | 32 (20%) | 0 (0%) | 0 (0%) | 1 (4%) |
| Weak (1-49%) | 42 (13%) | 23 (17%) | 56 (36%) | 0 (0%) | 0 (0%) | 7 (30%) |
| Strong (50-100%) | 56 (18%) | 22 (17%) | 21 (13%) | 0 (0%) | 0 (0%) | 1 (4%) |
| Unknown | 0 (0%) | 0 (0%) | 48 (31%) | 335 (100%) | 952 (100%) | 14 (61%) |
| **Therapy** |  |  |  |  |  |  |
| Anti-PD-(L)1 monotherapy | 281 (90%) | 122 (92%) | 59(38%) | 317(95%) | - | 0 (0%) |
| Anti-PD-(L)1 in combination with anti-CTLA-4 | 32 (10%) | 11 (8.3%) | 75(48%) | 18(5%) | - | 0 (0%) |
| Anti-PD-(L)1 in combination with chemotherapy | 0 (0%) | 0 (0%) | 23(15%) | 0 (0%) | - | 23 (100%) |
| **Clinical benefit** |  |  |  |  |  |  |
| DCB | 98 (31%) | 42 (32%) | 74 (47%) | - | - | 12 (52%) |
| NDB | 215 (69%) | 91 (68%) | 64 (41%) | - | - | 9 (39%) |
| Unassessable | 0 (0%) | 0 (0%) | 19 (12%) | - | - | 2 (9%) |
| **BOR** |  |  |  |  |  |  |
| CR/PR | 66 (21%) | 25 (19%) | 43 (27%) | - | - | 0 (0%) |
| SD/PD | 247 (79%) | 108 (81%) | 84 (54%) | - | - | 0 (0%) |
| Unassessable | 0 (0%) | 0 (0%) | 30 (19%) | - | - | 23 (100%) |
| **Abbreviations:** The Cancer Genome Atlas, TCGA; overall survival, OS; anti-programmed cell death 1 (ligand 1), anti-PD-(L)1; anti-cytotoxic T-cell lymphocyte-4, anti-CTLA-4; durable clinical benefit, DCB; no durable benefit, NDB; best overall response, BOR; complete response, CR; partial response, PR; stable disease, SD; progressive disease, PD. | | | | | | |

**Table S3. X-squared test of the mutate genes between durable clinical benefit (DCB) and no durable benefit (NDB).**

| **Variable** | **X-squared** | **p-value** |
| --- | --- | --- |
| PTPRT | 12.418 | 0.001 |
| EGFR | 8.174 | 0.002 |
| EPHA3 | 10.007 | 0.002 |
| ERBB4 | 8.872 | 0.002 |
| ATRX | 7.793 | 0.006 |
| PTPRD | 7.116 | 0.007 |
| AMER1 | 6.465 | 0.013 |
| TERT | 5.206 | 0.020 |
| HGF | 5.172 | 0.022 |
| POLE | 5.42 | 0.022 |
| STK11 | 5.461 | 0.023 |
| NTRK3 | 4.864 | 0.032 |
| EPHA5 | 4.325 | 0.037 |
| ALK | 4.44 | 0.040 |
| PGR | 4.509 | 0.041 |
| PIK3C2G | 3.721 | 0.053 |
| FAT1 | 3.361 | 0.064 |
| BCOR | 3.367 | 0.073 |
| KMT2D | 3.042 | 0.096 |
| MGA | 2.768 | 0.118 |
| PBRM1 | 2.207 | 0.153 |
| ARID1A | 2.127 | 0.158 |
| NFE2L2 | 2.125 | 0.158 |
| MDC1 | 2.457 | 0.172 |
| MLL3 | 1.824 | 0.205 |
| KRAS | 1.383 | 0.240 |
| ARID2 | 1.167 | 0.287 |
| PDGFRA | 1.167 | 0.287 |
| TP53 | 1.166 | 0.294 |
| PAK7 | 0.974 | 0.346 |
| TBX3 | 0.974 | 0.346 |
| ATM | 0.992 | 0.365 |
| KEAP1 | 0.86 | 0.389 |
| NOTCH4 | 0.829 | 0.420 |
| MET | 0.675 | 0.502 |
| PIK3CA | 0.672 | 0.520 |
| ERBB2 | 0.566 | 0.542 |
| GRIN2A | 0.566 | 0.542 |
| NOTCH3 | 0.274 | 0.627 |
| NF1 | 0.408 | 0.638 |
| APC | 0.259 | 0.683 |
| PIK3CG | 0.259 | 0.683 |
| RB1 | 0.259 | 0.697 |
| RBM10 | 0.233 | 0.745 |
| MLL2 | 0.13 | 0.821 |
| CREBBP | 0.133 | 0.827 |
| ZFHX3 | 0.05 | 0.833 |
| CDKN2A | 0 | 1 |
| SETD2 | 0.012 | 1 |
| SMARCA4 | 0.002 | 1 |

**Table S4. Gene numbers of each module in weighted gene co-expression network analysis (WGCNA) analysis.**

| **Module number** | **Module Colors** | **Genes** |
| --- | --- | --- |
| 1 | black | 97 |
| 2 | blue | 422 |
| 3 | brown | 356 |
| 4 | green | 202 |
| 5 | grey | 2957 |
| 6 | pink | 56 |
| 7 | red | 107 |
| 8 | turquoise | 543 |
| 9 | yellow | 260 |

**Table S5. Tumor infiltrating immune cells comparison between high- and low-score groups using** **CIBERSORTx.**

| **Variable** | **High, N = 280^1^** | **Low, N = 564^1^** | **p-value^2^** |
| --- | --- | --- | --- |
| B cells naive | 0.016 (0.004, 0.035) | 0.015 (0.005, 0.033) | 0.2851 |
| B cells memory | 0.000 (0.000, 0.000) | 0.000 (0.000, 0.000) | 0.9926 |
| Plasma cells | 0.08 (0.03, 0.13) | 0.09 (0.03, 0.14) | 0.7908 |
| T cells CD8 | 0.07 (0.03, 0.13) | 0.06 (0.03, 0.11) | **0.0236** |
| T cells CD4 naive | 0.000 (0.000, 0.000) | 0.000 (0.000, 0.000) | 0.3752 |
| T cells CD4 memory resting | 0.10 (0.06, 0.15) | 0.12 (0.07, 0.17) | 0.9937 |
| T cells CD4 memory activated | 0.02 (0.00, 0.06) | 0.01 (0.00, 0.05) | **0.0079** |
| T cells follicular helper | 0.021 (0.003, 0.039) | 0.016 (0.000, 0.033) | **0.0066** |
| T cells gamma delta | 0.000 (0.000, 0.014) | 0.000 (0.000, 0.006) | 0.8345 |
| T cells regulatory (Tregs) | 0.018 (0.001, 0.036) | 0.018 (0.005, 0.040) | 0.0533 |
| NK cells resting | 0.000 (0.000, 0.011) | 0.000 (0.000, 0.012) | 0.1976 |
| NK cells activated | 0.018 (0.000, 0.037) | 0.014 (0.000, 0.030) | **0.0322** |
| Monocytes | 0.000 (0.000, 0.009) | 0.000 (0.000, 0.009) | 0.8914 |
| Macrophages M0 | 0.16 (0.09, 0.26) | 0.16 (0.09, 0.28) | 0.5924 |
| Macrophages M1 | 0.08 (0.05, 0.11) | 0.07 (0.04, 0.11) | **0.0117** |
| Macrophages M2 | 0.14 (0.10, 0.19) | 0.14 (0.10, 0.19) | 0.4795 |
| Dendritic cells resting | 0.02 (0.00, 0.06) | 0.03 (0.01, 0.07) | 0.9571 |
| Dendritic cells activated | 0.000 (0.000, 0.022) | 0.003 (0.000, 0.027) | 0.9792 |
| Mast cells resting | 0.04 (0.02, 0.07) | 0.04 (0.02, 0.07) | 0.7022 |
| Mast cells activated | 0.000 (0.000, 0.000) | 0.000 (0.000, 0.000) | 0.9028 |
| Eosinophils | 0.0000 (0.0000, 0.0000) | 0.0000 (0.0000, 0.0000) | 0.8283 |
| Neutrophils | 0.003 (0.000, 0.010) | 0.004 (0.000, 0.012) | 0.9700 |
| **^1^Median (IQR)**  **^2^Wilcoxon rank sum test** | | | |

**Table S6. Tumor infiltrating immune cell subtypes comparison between high- and low-score groups using Ecotyper.**

| **Variable** | **Overall, N = 844** | **High, N = 280** | **Low, N = 564** | **p-value^1^** |
| --- | --- | --- | --- | --- |
| **CD8 T cell** |  |  |  | **0.0015** |
| S01 | 200 (56%) | 49 (47%) | 151 (59%) |  |
| S02 | 87 (24%) | 22 (21%) | 65 (26%) |  |
| S03 | 71 (20%) | 33 (32%) | 38 (15%) |  |
| **CD4 T cell** |  |  |  | **0.0083** |
| S01 | 85 (26%) | 28 (27%) | 57 (25%) |  |
| S02 | 98 (30%) | 24 (23%) | 74 (33%) |  |
| S03 | 11 (3.3%) | 6 (5.7%) | 5 (2.2%) |  |
| S04 | 51 (15%) | 10 (9.5%) | 41 (18%) |  |
| S05 | 38 (11%) | 12 (11%) | 26 (11%) |  |
| S06 | 33 (9.9%) | 16 (15%) | 17 (7.5%) |  |
| S07 | 16 (4.8%) | 9 (8.6%) | 7 (3.1%) |  |
| **B Cell** |  |  |  | **0.0003** |
| S01 | 124 (26%) | 28 (20%) | 96 (29%) |  |
| S02 | 54 (11%) | 26 (18%) | 28 (8.4%) |  |
| S03 | 94 (20%) | 20 (14%) | 74 (22%) |  |
| S04 | 35 (7.3%) | 17 (12%) | 18 (5.4%) |  |
| S05 | 170 (36%) | 51 (36%) | 119 (36%) |  |
| **Dendritic cell** |  |  |  | 0.3311 |
| S01 | 132 (37%) | 30 (30%) | 102 (40%) |  |
| S02 | 40 (11%) | 10 (10%) | 30 (12%) |  |
| S03 | 42 (12%) | 17 (17%) | 25 (9.8%) |  |
| S04 | 9 (2.5%) | 4 (4.0%) | 5 (2.0%) |  |
| S05 | 40 (11%) | 9 (9.0%) | 31 (12%) |  |
| S06 | 17 (4.8%) | 5 (5.0%) | 12 (4.7%) |  |
| S07 | 38 (11%) | 13 (13%) | 25 (9.8%) |  |
| S08 | 38 (11%) | 12 (12%) | 26 (10%) |  |
| **Endothelial cell** |  |  |  | **0.0002** |
| S01 | 35 (5.5%) | 14 (7.2%) | 21 (4.8%) |  |
| S02 | 228 (36%) | 72 (37%) | 156 (35%) |  |
| S03 | 183 (29%) | 35 (18%) | 148 (34%) |  |
| S04 | 152 (24%) | 63 (32%) | 89 (20%) |  |
| S05 | 37 (5.8%) | 10 (5.2%) | 27 (6.1%) |  |
| **Epithelial cell** |  |  |  | **0.0007** |
| S01 | 252 (32%) | 74 (29%) | 178 (33%) |  |
| S02 | 82 (10%) | 18 (7.2%) | 64 (12%) |  |
| S03 | 80 (10%) | 21 (8.4%) | 59 (11%) |  |
| S04 | 79 (10.0%) | 31 (12%) | 48 (8.9%) |  |
| S05 | 186 (23%) | 53 (21%) | 133 (25%) |  |
| S06 | 114 (14%) | 54 (22%) | 60 (11%) |  |
| **Fibroblast** |  |  |  | **0.0048** |
| S01 | 7 (1.1%) | 4 (2.1%) | 3 (0.7%) |  |
| S02 | 86 (14%) | 19 (10%) | 67 (16%) |  |
| S03 | 86 (14%) | 20 (11%) | 66 (16%) |  |
| S04 | 12 (2.0%) | 5 (2.6%) | 7 (1.7%) |  |
| S05 | 82 (13%) | 28 (15%) | 54 (13%) |  |
| S06 | 95 (16%) | 41 (22%) | 54 (13%) |  |
| S07 | 86 (14%) | 18 (9.5%) | 68 (16%) |  |
| S08 | 157 (26%) | 54 (29%) | 103 (24%) |  |
| **Mast cell** |  |  |  | **0.0001** |
| S01 | 55 (9.9%) | 14 (8.6%) | 41 (10%) |  |
| S02 | 123 (22%) | 24 (15%) | 99 (25%) |  |
| S03 | 84 (15%) | 41 (25%) | 43 (11%) |  |
| S04 | 87 (16%) | 20 (12%) | 67 (17%) |  |
| S05 | 149 (27%) | 41 (25%) | 108 (27%) |  |
| S06 | 60 (11%) | 22 (14%) | 38 (9.6%) |  |
| **Monocyte and Macrophage** |  |  |  | 0.0675 |
| S01 | 66 (11%) | 14 (7.7%) | 52 (13%) |  |
| S02 | 117 (20%) | 27 (15%) | 90 (23%) |  |
| S03 | 106 (18%) | 38 (21%) | 68 (17%) |  |
| S04 | 47 (8.1%) | 14 (7.7%) | 33 (8.3%) |  |
| S05 | 24 (4.1%) | 7 (3.8%) | 17 (4.3%) |  |
| S06 | 71 (12%) | 22 (12%) | 49 (12%) |  |
| S07 | 51 (8.8%) | 23 (13%) | 28 (7.1%) |  |
| S08 | 56 (9.7%) | 22 (12%) | 34 (8.6%) |  |
| S09 | 41 (7.1%) | 15 (8.2%) | 26 (6.5%) |  |
| **NK cell** |  |  |  | **0.0120** |
| S01 | 147 (32%) | 47 (35%) | 100 (31%) |  |
| S02 | 122 (27%) | 33 (25%) | 89 (28%) |  |
| S03 | 109 (24%) | 22 (17%) | 87 (27%) |  |
| S04 | 46 (10%) | 22 (17%) | 24 (7.4%) |  |
| S05 | 32 (7.0%) | 9 (6.8%) | 23 (7.1%) |  |
| **Plasma cell** |  |  |  | **0.0365** |
| S01 | 85 (24%) | 22 (18%) | 63 (27%) |  |
| S02 | 125 (35%) | 40 (33%) | 85 (36%) |  |
| S03 | 54 (15%) | 24 (20%) | 30 (13%) |  |
| S04 | 19 (5.3%) | 5 (4.1%) | 14 (6.0%) |  |
| S05 | 34 (9.5%) | 11 (8.9%) | 23 (9.8%) |  |
| S06 | 40 (11%) | 21 (17%) | 19 (8.1%) |  |
| **^1^Chi-square test** | | | | |

**Table S7. Overview of cell states.**

| **Cell type** | **Cell state** | **Key marker genes** | **Cell state annotation** | **Carcinoma ecotype** |
| --- | --- | --- | --- | --- |
|  |  |  |  |  |
| B cells | S01 | MS4A1, TCL1A | Classical naïve (S01) | CE10 |
| B cells | S02 | FXYD7 | Unknown (S02) | CE5 |
| B cells | S03 | PPP1R12B | Normal-enriched (S03) | CE6 |
| B cells | S04 | IKBKB | Activated (S04) | CE8 |
| B cells | S05 | CD53, SASH3 | Activated (S05) | CE9 |
| CD4 T cells | S01 | CXCR6, CTLA4 | Exhausted/effector memory/Treg (S01) | CE9 |
| CD4 T cells | S02 | CCR7 | Naïve/central memory (S02) | CE10 |
| CD4 T cells | S03 | GLYCTK | Unknown (S03) | NA |
| CD4 T cells | S04 | KLF2 | Resting (normal-enriched) (S04) | CE6 |
| CD4 T cells | S05 | ARHGEF9 | Unknown (S05) | NA |
| CD4 T cells | S06 | ANKIB1 | Unknown (S06) | CE7 |
| CD4 T cells | S07 | LRRC69 | Unknown (S07) | CE8 |
| CD8 T cells | S01 | BTLA, GZMK | Naïve/central memory (S01) | CE10 |
| CD8 T cells | S02 | FCGR3A, LAIR1 | Late-stage differentiated effector (S02) | CE3 |
| CD8 T cells | S03 | IFNG, GZMB, LAG3 | Exhausted/effector memory (S03) | CE9 |
| Dendritic cells | S01 | CLEC9A, XCR1 | Myeloid cDC1 (S01) | CE10 |
| Dendritic cells | S02 | ITGAM, CLEC12A | Myeloid cDC2-B (Inflammatory) (S02) | CE3 |
| Dendritic cells | S03 | PRF1, CD274, CD80 | Mature immunogenic (S03) | CE9 |
| Dendritic cells | S04 | AKAP6 | Unknown (S04) | CE4 |
| Dendritic cells | S05 | CAV1 | Mature (normal-enriched) (S05) | CE6 |
| Dendritic cells | S06 | CD207, CD1A | Langerhans-like (S06) | NA |
| Dendritic cells | S07 | CXCL2, CXCL8 | Migratory activated (S07) | CE2 |
| Dendritic cells | S08 | PDPN | Unknown (S08) | CE1 |
| Endothelial cells | S01 | CD36 | Normal-enriched (S01) | CE6 |
| Endothelial cells | S02 | ANGPTL2, NID2 | Tumor-associated/tip cells (S02) | CE1 |
| Endothelial cells | S04 | ITGA3 | Unknown (S04) | CE8 |
| Endothelial cells | S05 | CNKSR1 | Myoendothelium-like (S05) | CE4 |
| Epithelial cells | S01 | KRT6A | Basal-like (S01) | CE2 |
| Epithelial cells | S02 | CITED2 | Normal-enriched (S02) | CE6 |
| Epithelial cells | S03 | ITGA3 | Pro-angiogenic (S03) | CE1 |
| Epithelial cells | S04 | GBP1, IRF1 | Pro-inflammatory (S04) | CE9 |
| Epithelial cells | S05 | AGR2 | Unknown (S05) | NA |
| Epithelial cells | S06 | CBX1 | Metabolic (S06) | CE8 |
| Fibroblasts | S01 | FITM1, CDH15 | Myofibroblast-like (S01) | CE4 |
| Fibroblasts | S02 | CD34, SPARCL1 | CAF2 (normal-enriched) (S02) | CE6 |
| Fibroblasts | S03 | POSTN, COL10A1 | CAF1 (tumor-associated) (S03) | CE1 |
| Fibroblasts | S04 | PUM1, FGFR1OP2 | Migratory-like (S04) | CE7 |
| Fibroblasts | S05 | PPP1R32 | Unknown (S05) | CE8 |
| Fibroblasts | S06 | SLC29A4 | Unknown (S06) | CE5 |
| Fibroblasts | S08 | CA9 | Pro-migratory-like (S08) | CE2 |
| Mast cells | S01 | CMA1 | Normal-enriched (S01) | CE6 |
| Mast cells | S02 | NAPSA | Normal-enriched (S02) | NA |
| Mast cells | S03 | GPATCH2 | Unknown (S03) | CE8 |
| Mast cells | S04 | TPSAB1 | Classical (S04) | CE10 |
| Mast cells | S05 | ARRB2 | Unknown (S05) | CE1 |
| Mast cells | S06 | IL1B | Activated (S06) | CE3 |
| Monocytes/Macrophages | S01 | CCR2, CLEC10A | Monocytes (S01) | CE10 |
| Monocytes/Macrophages | S02 | FABP4, MARCO | Classical M0 (normal-enriched) (S02) | NA |
| Monocytes/Macrophages | S03 | CXCL9, SLAMF8 | Classical M1 (S03) | CE9 |
| Monocytes/Macrophages | S04 | CD300E, CLEC5A | Classical M2 (S04) | CE3 |
| Monocytes/Macrophages | S05 | S1PR1 | M2-like (normal-enriched) (S05) | CE6 |
| Monocytes/Macrophages | S06 | AEBP1 | M2 foam cell-like (S06) | CE1 |
| Monocytes/Macrophages | S07 | CHI3L2 | M2-like proliferative (S07) | CE2 |
| Monocytes/Macrophages | S08 | CDK4 | Proliferative (S08) | CE7 |
| Monocytes/Macrophages | S09 | DLEC1 | Unknown (S09) | CE8 |
| NK cells | S01 | PRF1, CD247 | Classical (S01) | CE9 |
| NK cells | S02 | STX11 | Normal-enriched (S02) | NA |
| NK cells | S03 | CD59 | Unknown (S03) | CE1 |
| NK cells | S04 | VAC14 | Unknown (S04) | NA |
| NK cells | S05 | CARNS1 | Unknown (S05) | CE5 |
| Plasma cells | S01 | CD27 | Classical (S01) | CE10 |
| Plasma cells | S02 | CXCR4 | Unknown (S02) | CE9 |
| Plasma cells | S03 | ASCL1 | Unknown (S03) | CE5 |
| Plasma cells | S04 | DHRS11 | Unknown (S04) | NA |
| Plasma cells | S05 | ITGA8 | Normal-enriched (S05) | NA |
| Plasma cells | S06 | ING1 | Unknown (S06) | CE7 |

**Supplementary** **figure S1**

**
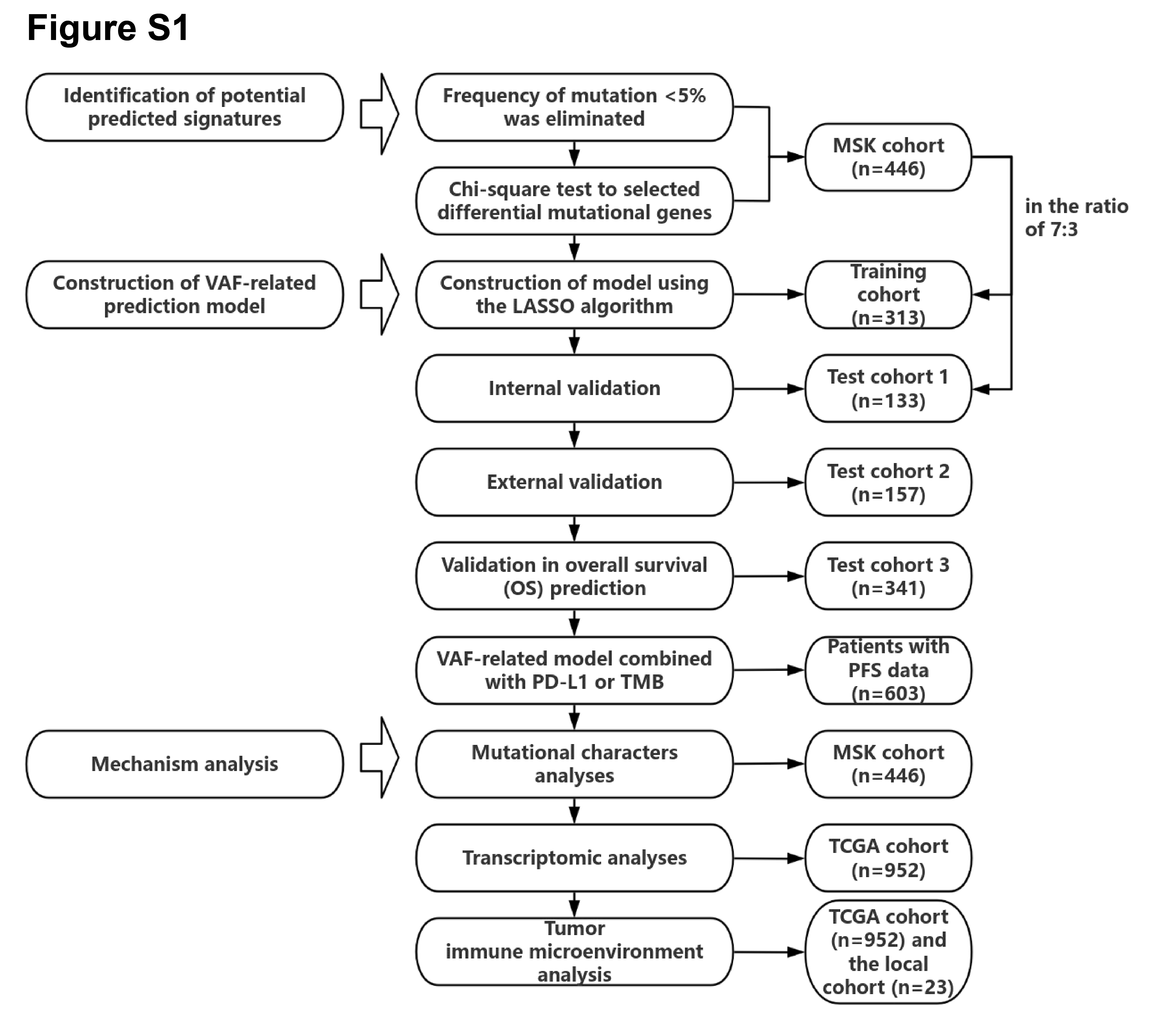
**

**Figure S1. The workflow of this study.**

**(A)** The workflow of this study.

**Supplementary figure S2**

**
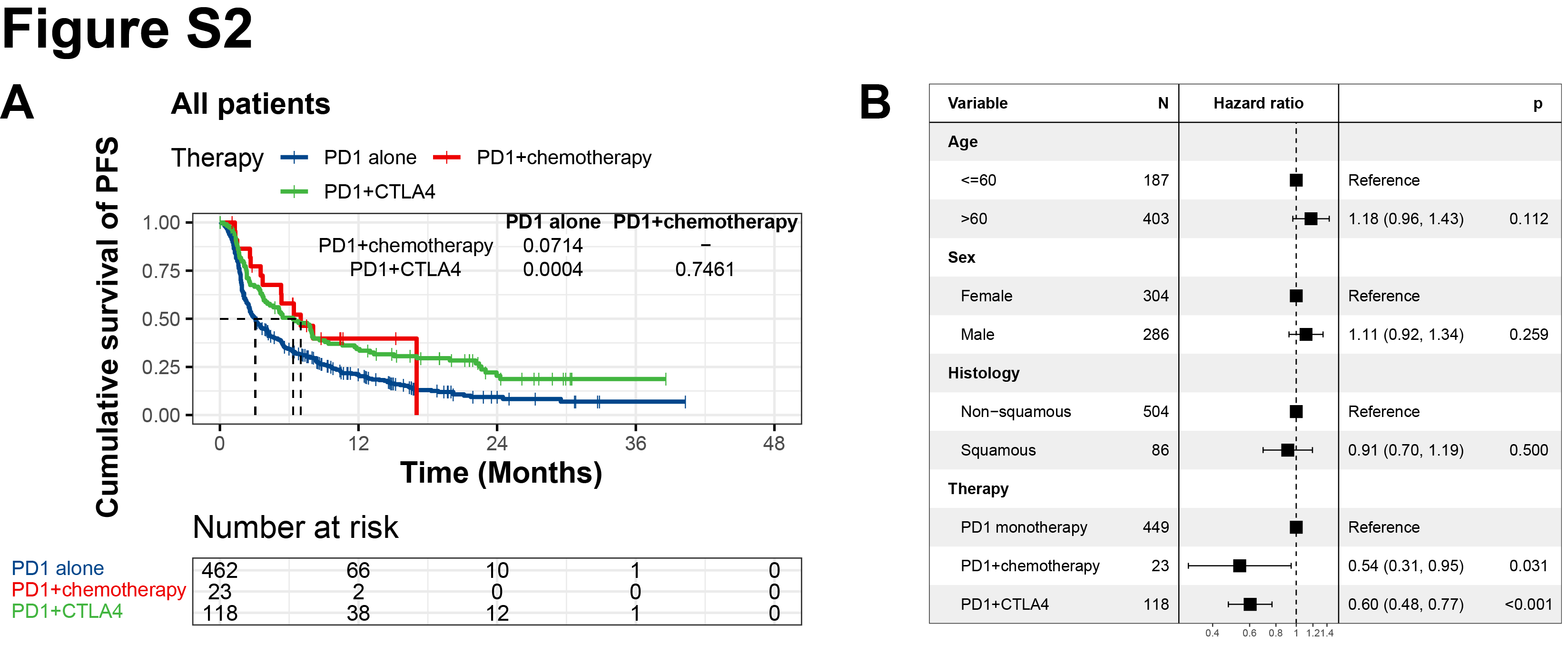
**

**Figure S2. Subgroup analysis of different** **therapy types.**

**(A)** Kaplan-Meier (K-M) survival curve of progression-free survival (PFS) in different therapy types. **(B)** Multivariate Cox regression analysis of therapy types and other clinical characteristics.

**Supplementary figure S3**

**
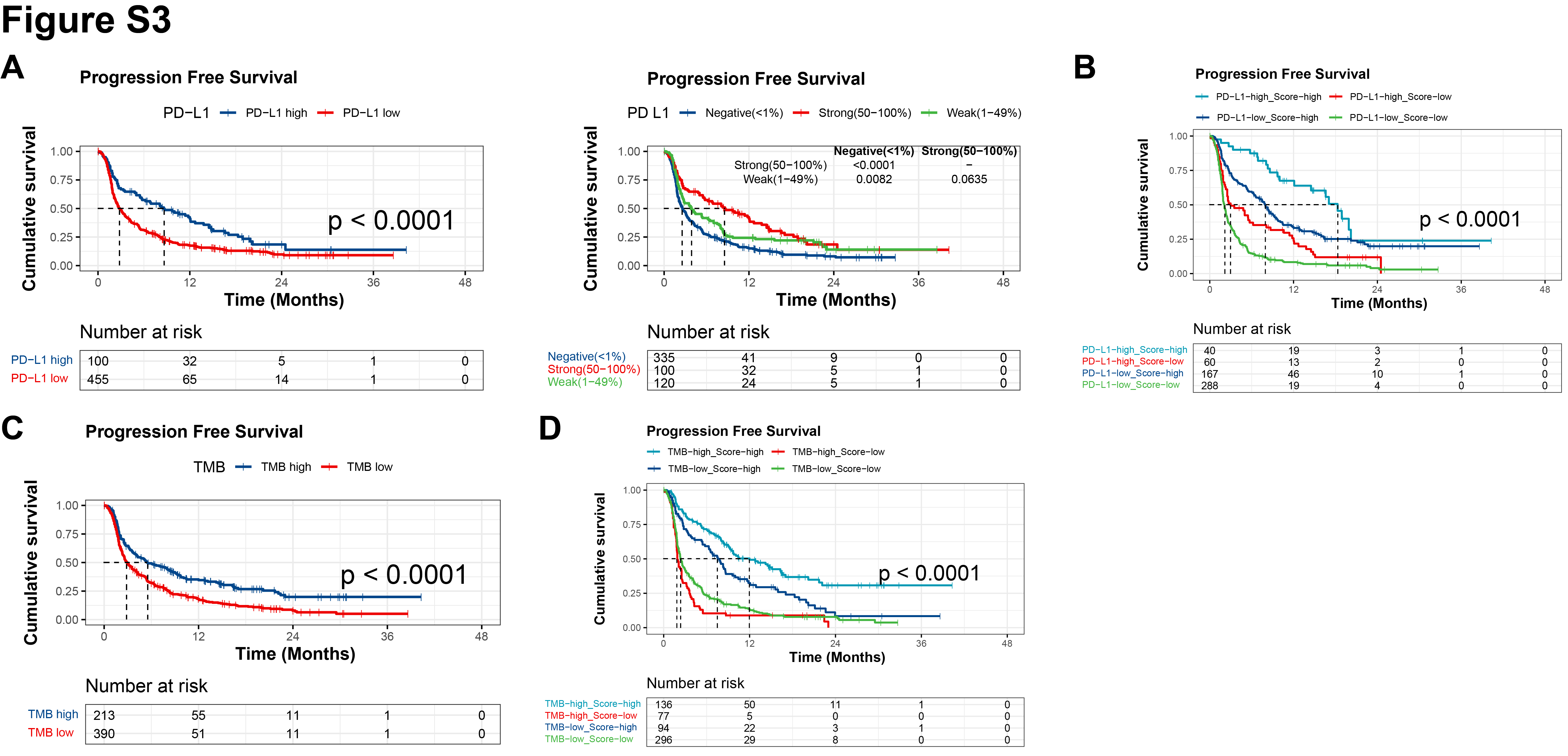
**

**Figure S3. Kaplan-Meier (K-M) survival curve of progression free survival (PFS) for PD-L1 and TMB alone or in combination with the VAF-related model .**

**(A)** K-M survival curve of PFS for PD-L1 alone. **(B)** K-M survival curve of PFS for PD-L1 in combination with Riskscore. PD-L1 <1% was considered PD-L1 low, and 1%-100% was considered PD-L1 high. PD-L1 <1% was negative, 1-49% was weak, and 50%-100% was strong. **(C)** K-M survival curve of PFS for TMB alone. **(D)** K-M survival curve of PFS for TMB in combination with Riskscore. TMB <10 mut/Mb was considered TMB low, and >10 mut/Mb was considered TMB high. P-value was calculated from log-rank test.

**Supplementary figure S4**


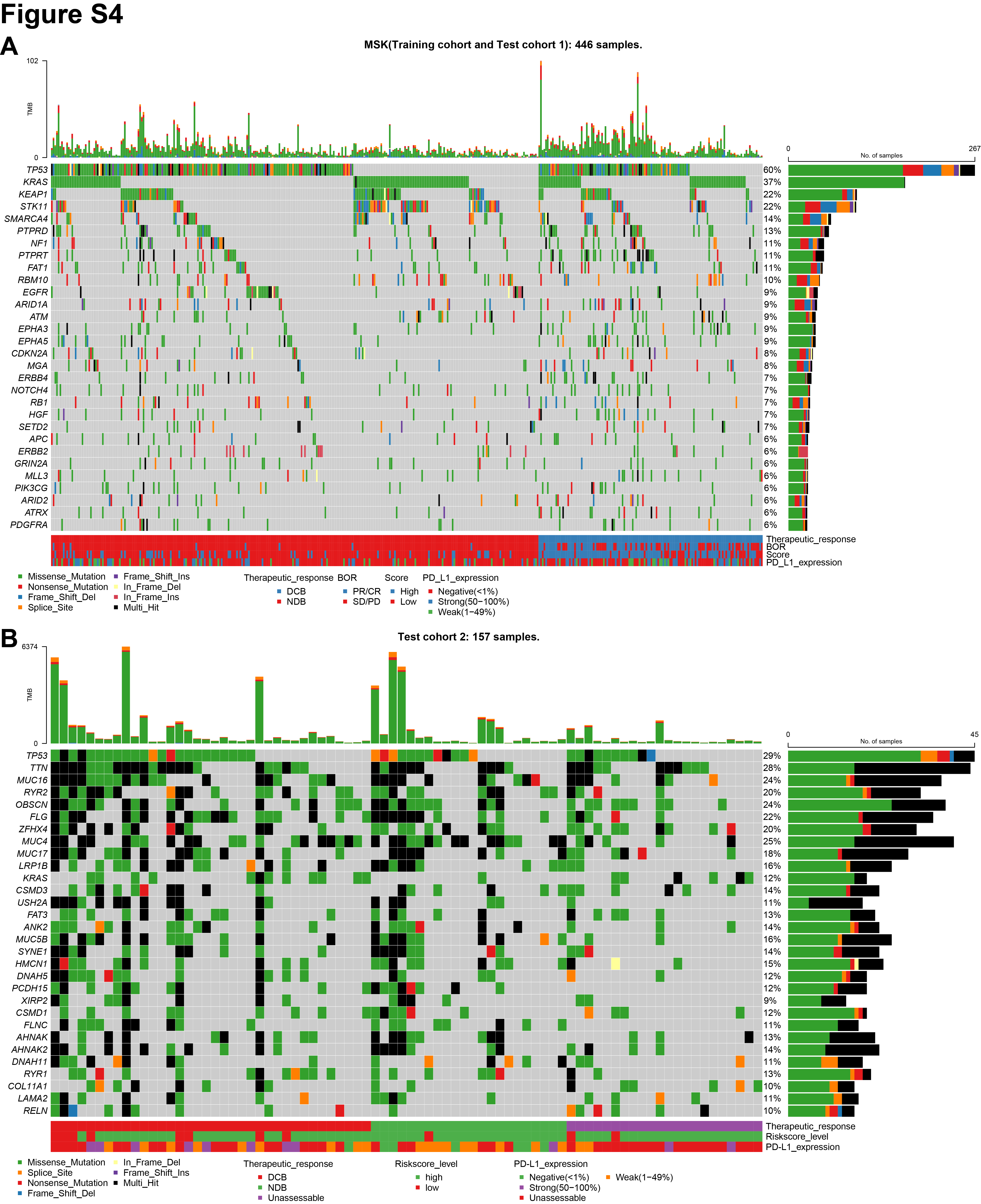


**Figure S4. Oncoplot for top 30 mutated genes of the cohorts.**

**(A)** Oncoplot for top 30 mutated genes of MSK cohort (Training cohort and Test cohort 1) (446 samples). **(B)** Oncoplot fro top 30 mutated genes of Test cohort 2 (157 samples).

**Supplementary figure S5**


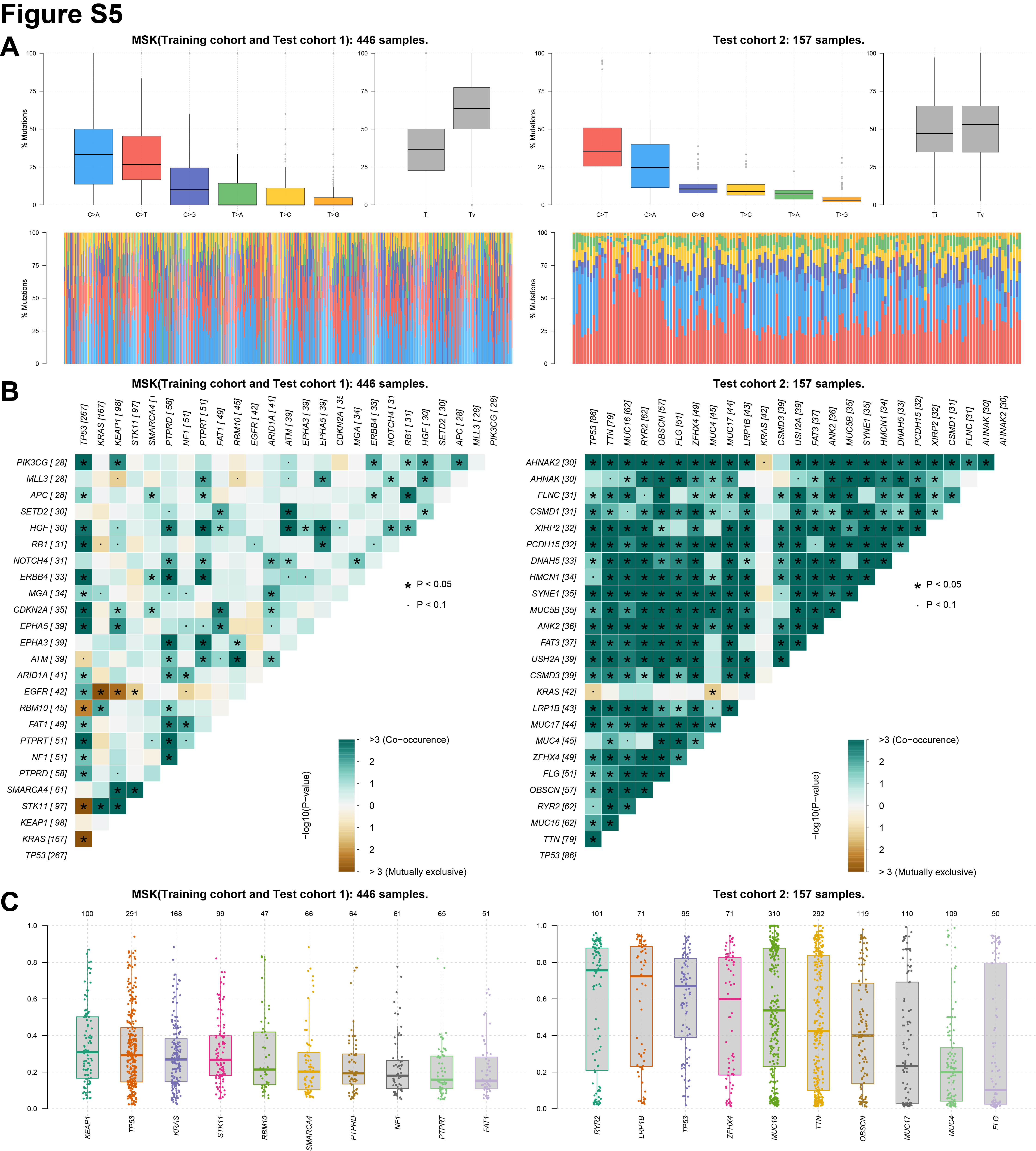


**Figure S5. Mutational analyses of the MSK cohort and Test cohort 2.**

**(A)** Transition and transversions analysis of the cohorts. **(B)** Somatic interactions analysis of the cohorts. **(C)** Variant Allele Frequency (VAF) visualization of the cohorts.

**Supplementary figure S6**


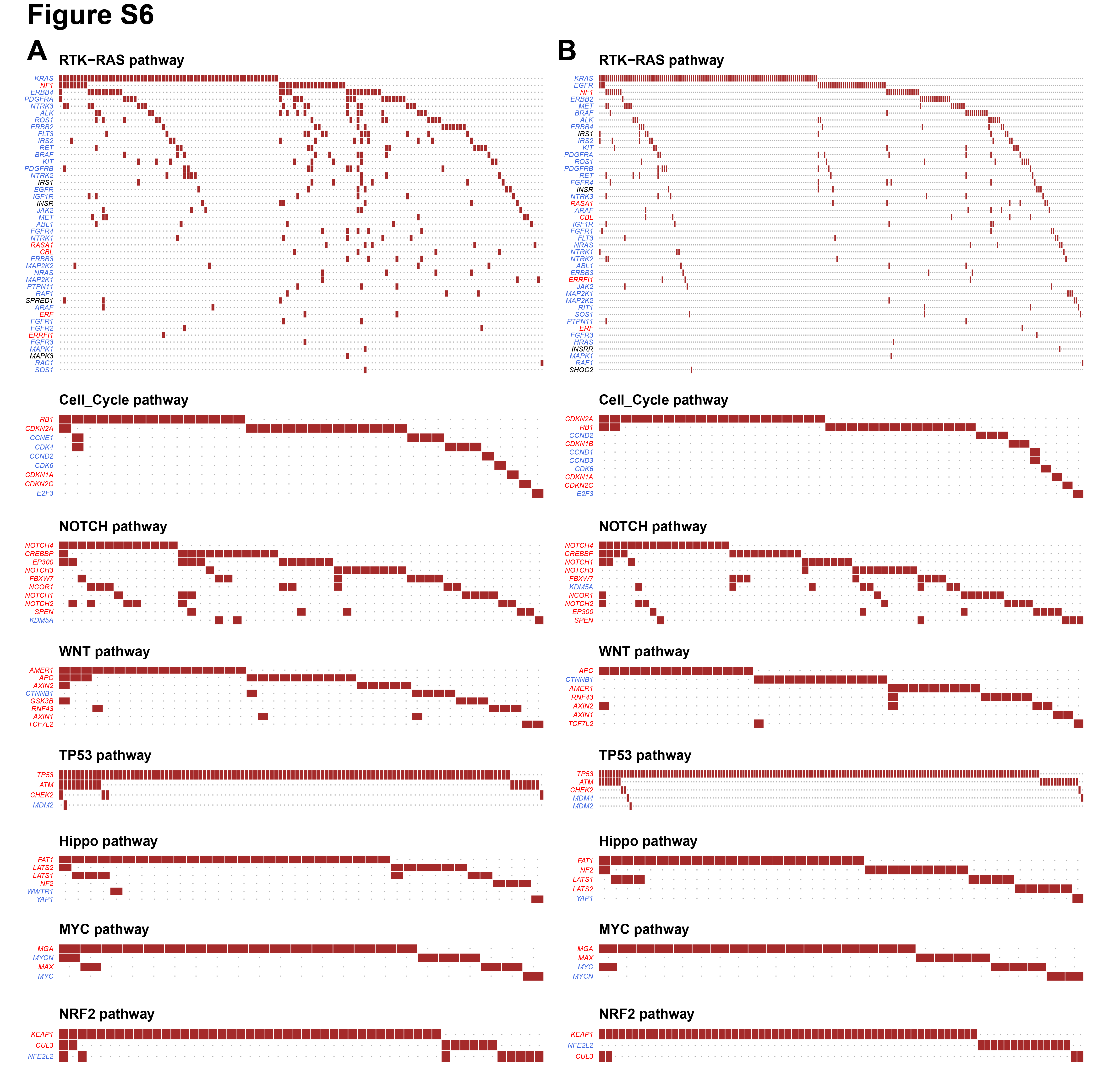


**Figure S6.** **Complete oncogenic signaling pathways visualization of the high- and low- score groups.**

**(A)** Oncogenic signaling pathways of the high-score group. **(B)** Oncogenic signaling pathways of the low-score group.

**Supplementary figure S7**


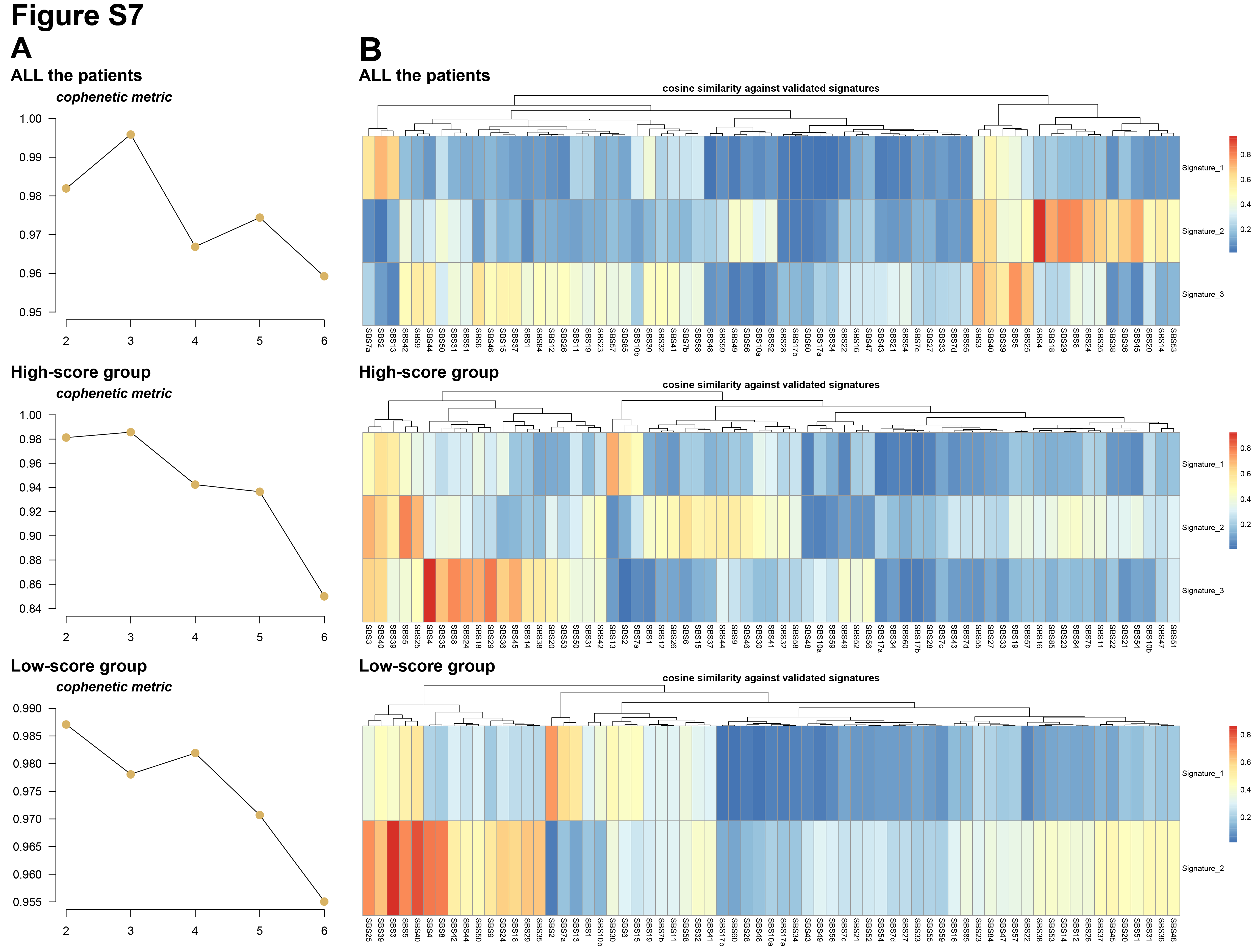


**Figure S7.** **Single-base substitution (SBS) signature analysis.**

**(A)** Elblow plot to visualize and decide optimal number of signatures. Best possible signature is the value at which cophenetic correlation drops significantly. All the patients (n=3). High-score group (n=3). Low-score group (n=2). **(B)** Comparison of similarities of extracted signatures against 60 validated signatures from COSMIC database, in all the patients, high-score group, low-score group, respectively.

**Supplementary figure S8**


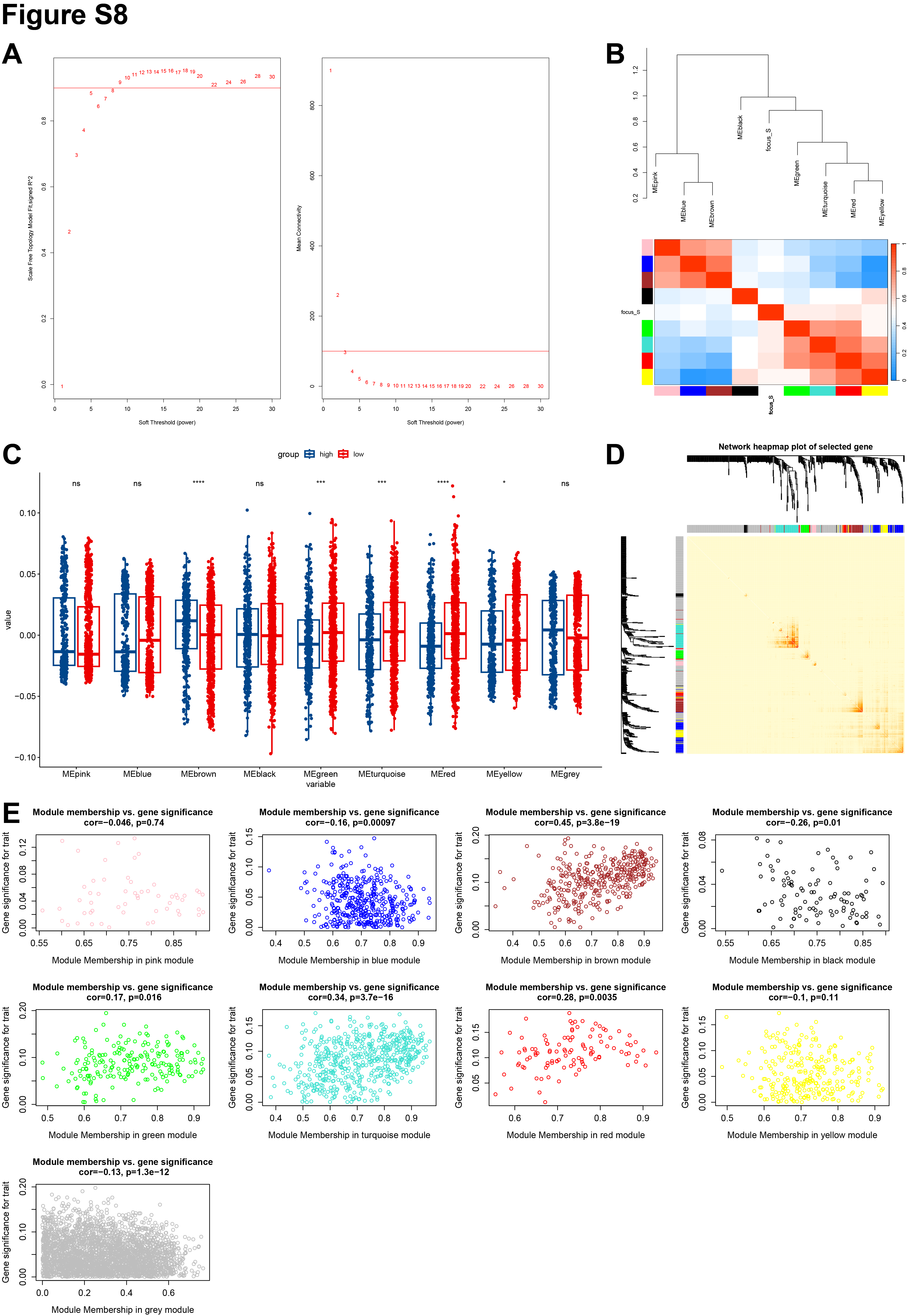


**Figure S8. Weighted gene co-expression network analysis (WGCNA).**

**(A)** Pick soft threshold. We picked the first number which the scale free topology model fit reached 0.9 as soft threshold (power = 9). **(B)** Hierarchical clustering and heatmap plot of the modules. **(C)** Boxplots of the traits cpmparison between the modules. **(D)** Heatmap plot of topological overlap in the gene network. The gene dendrogram and module assignment are shown along the left and top. **(E)** Scatterplots of gene significance for weight (GS) versus module membership (MM) in the modules. A P-value < 0.05 was considered significant. Levels of significance: *: P < 0.05; **: P < 0.01; ***: P < 0.001; ****: P < 0.0001.

**Supplementary figure S9**


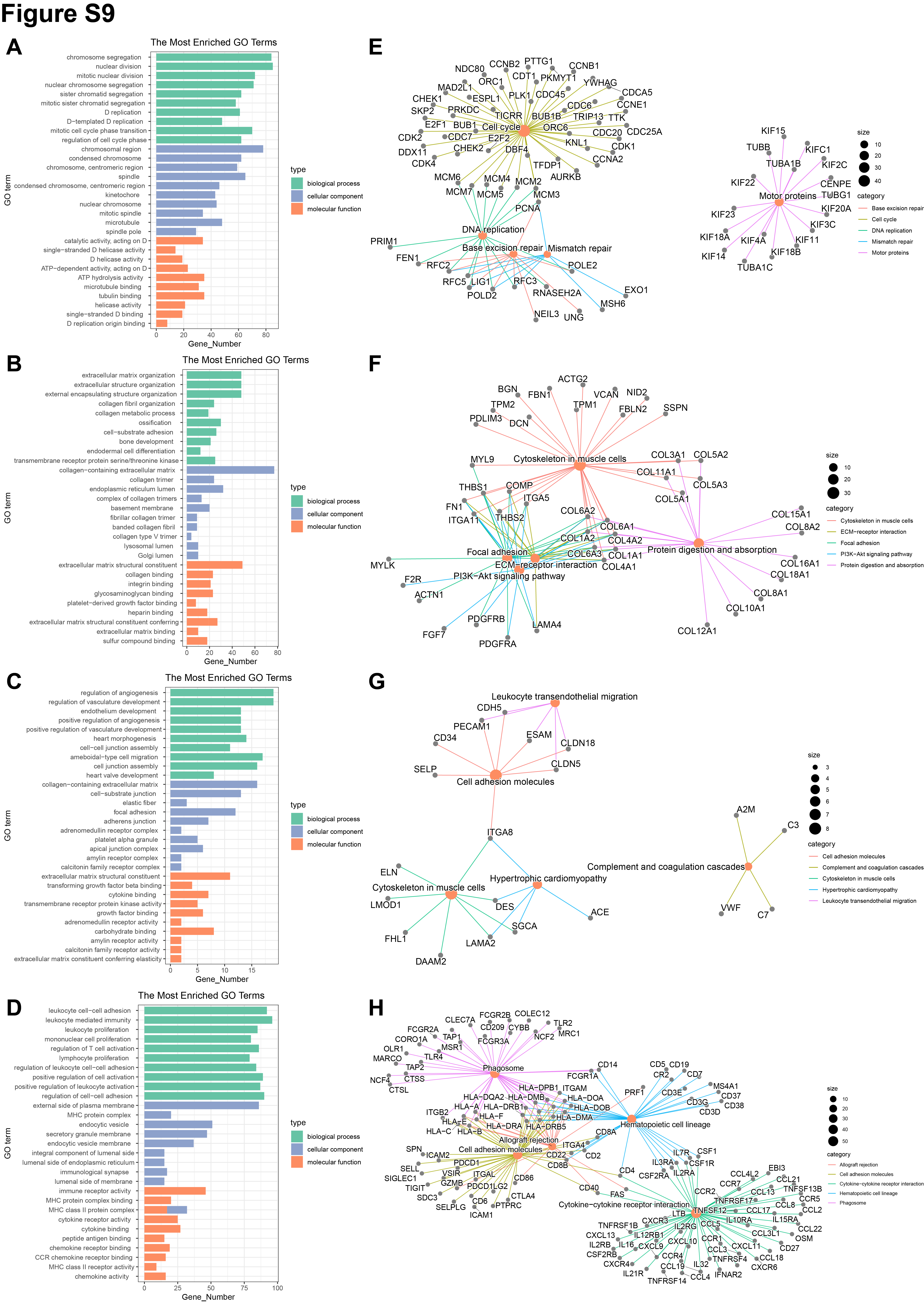


**Figure S9. Barplot and network map of** **the enrichment analysis in each module.**

**(A)-(D)** The top 10 pathways of Gene Ontology (GO) enrichment analysis in the brown, green, red, and turquoise module, respectively. **(E)-(H)** The top 5 pathways of [Kyoto encyclopedia of genes and genomes](https://pubmed.ncbi.nlm.nih.gov/10592173/) (KEGG) enrichment analysis in the brown, green, red, and turquoise module, respectively.

**Supplementary figure S10**


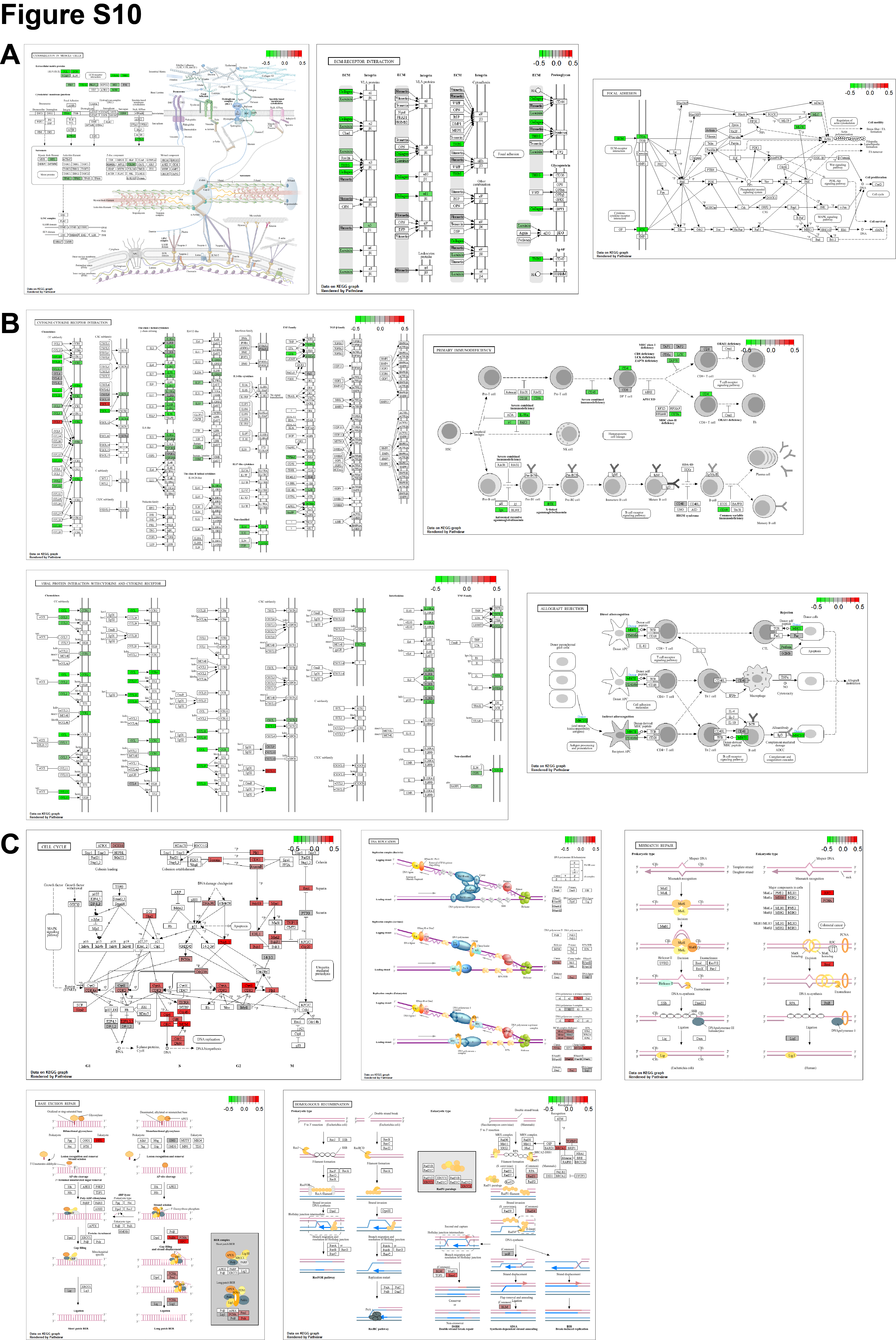


**Figure S10. Visualization of KEGG pathways.**

**(A)** Cellular interaction pathways (cytoskeleton in muscle cells, ECM-receptor interaction, and focal adhesion) enriched in the low-score group (the green module of WGCNA). **(B)** Immune and inflammation related pathways (cytokine-cytokine receptor interaction, primary immunodeficiency, viral protein interaction with cytokine and cytokine receptor, and allograft rejection) enriched in the low-score group (the turquoise module of WGCNA). **(C)** DNA damage response (DDR) pathways (cell cycle, DNA replication, mismatch repair, base excision repair, and homologous recombination) enriched in the high-score group (the brown module of WGCNA). The color of the genes represent log2fold change (log2FC) in differentially analysis between the high- and low- score groups. The red genes were up-regulated in the high-score group. The green genes were down-regulated in the high-score group.

**Supplementary figure S11**


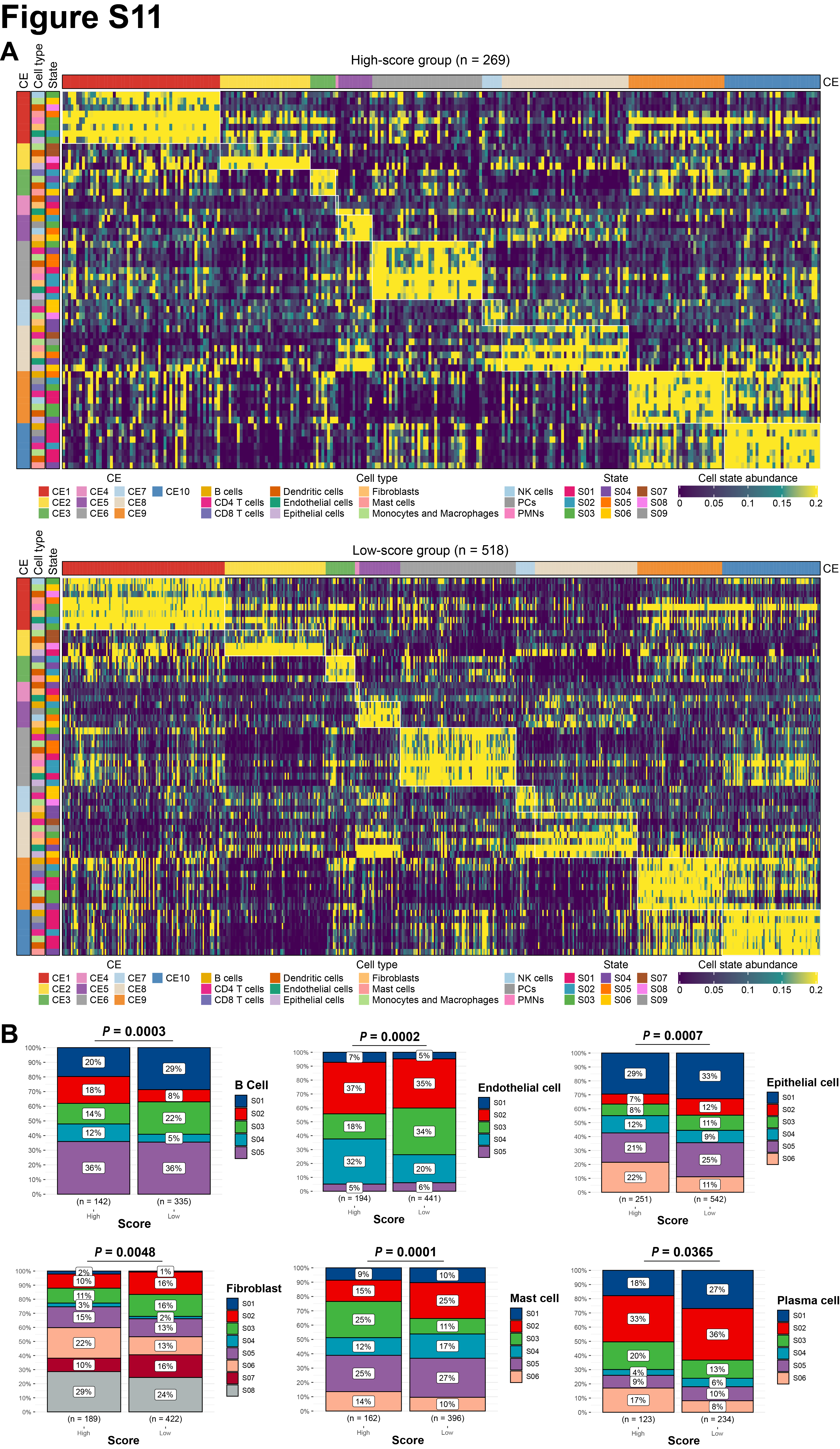


**Figure S11. Tumor immune microenvironment analysis using Ecotyper.**

**(A)** Heatmap of carcinoma ecotypes in the high- and low-score, respectively. **(B)** Cell states of B cell, endothelial cell, epithelial cell, fibroblast, mast cell, and plasma cell between high- and low-score groups. [Chi-square test](javascript:;).
